# Supplementary material for: Spectral bias and task-model alignment explain generalization in kernel regression and infinitely wide neural networks
Source: Nat Commun. 2021 May 18;12:2914. doi: 10.1038/s41467-021-23103-1 (PMC8131612; doi:10.1038/s41467-021-23103-1)
Supplement: Supplementary file 1 — Supplementary Information [file 41467_2021_23103_MOESM1_ESM.pdf]

Supplementary Information for “Spectral Bias and Task-Model Alignment Explain Generalization in Kernel Regression and Infinitely Wide Neural Networks"

Abdulkadir Canatar [1,2] Blake Bordelon [2,3] Cengiz Pehlevan [2,3,*]

Contents

Supplementary Note 1 - Problem Setup **2**

Supplementary Note 2 - Replica Calculation **3**
Averaging over Quenched Disorder 4
Replica Symmetry and Saddle Point Equations 7
Generalization Error 10
Training Error 11
Expected Estimator and the Correlation Function 11

Supplementary Note 3 - White Band-limited RKHS Spectrum **15**
Connection to Random Matrices 17

Supplementary Note 4 - Rotation Invariant Kernels **18**
Gaussian kernel 22
Dot-Product Kernels and Neural Tangent Kernel 23
Approximate Scaling of Learning Curves 27

Supplementary Note 5 - Details of Numerical Experiments **29**
Calculating Kernel Spectrum for Spherical Data 29
Synthetic Data Experiments on Unit Sphere 29
Pure Target Functions 30
Target Function with Non-zero Weights and Synthetic Kernel Regression Experiments . . 30
Details of Neural Network Experiments 32
White Band-limited Kernel Experiments 32

Supplementary Note 6 - Notes on Spherical Harmonics **32**

[1]Department of Physics, Harvard University, Cambridge, MA 02138, USA.
[2]Center for Brain Science, Harvard University, Cambridge, MA 02138, USA.
[3]John A. Paulson School of Engineering and Applied Sciences, Harvard University, Cambridge, MA 02138, USA.
[*]Corresponding Author, E-mail: cpehlevan@g.harvard.edu

Supplementary Discussion **33**
Relation to Kernel Alignment Risk Estimator (KARE) 33
Relation to Kernel Alignment 34
Relation to Other Statistical Physics Approaches to Kernel Machines 34

Supplementary Note 1 - Problem Setup

A reproducing kernel Hilbert space [1] $\mathcal{H}$ living on $\mathcal{X} \subset \mathbb{R}^D$ is a subset of square integrable functions $L_2(\mathcal{X}, p)$ for measure p, equipped with an inner product $\langle \cdot, \cdot \rangle_{\mathcal{H}}$ and a kernel satisfying the following reproducing property:

$$\langle f(\cdot), K(\cdot, \mathbf{x}) \rangle_{\mathcal{H}} = f(\mathbf{x}), \quad \forall f(\cdot) \in \mathcal{H},\ \forall \mathbf{x} \in \mathcal{X}, \tag{1}$$

with $K(., \mathbf{x})$ is itself being an element of $\mathcal{H}$. We assume data is drawn from a finite measure with density $p(\mathbf{x})$ and that the kernel has finite trace on this measure $\int K(\mathbf{x}, \mathbf{x}) p(\mathbf{x}) d\mathbf{x} < \infty$. We introduce the integral operator $T_K : L_2(\mathcal{X}, p) \to L_2(\mathcal{X}, p)$ which is a linear map from functions to functions

$$T_K[f](\mathbf{x}') = \int p(\mathbf{x}) K(\mathbf{x}, \mathbf{x}') f(\mathbf{x}) d\mathbf{x}. \tag{2}$$

Mercer's Theorem allows the diagonalization of the kernel in terms of the eigenfunctions of T_K, $\{\phi_\rho(\mathbf{x})\}|_{\rho=0}^{\infty}$ where $T_K[\phi_\rho] = \eta_\rho \phi_\rho$ with $\eta_\rho \geq 0$ [1, 2, 3]. This set of eigenfunctions forms an orthonormal basis with respect to $L_2(\mathcal{X}, p)$. The resulting Mercer decomposition of the kernel is

$$K(\mathbf{x}, \mathbf{x}') = \sum_{\rho=0}^{\infty} \eta_\rho \phi_\rho(\mathbf{x}) \phi_\rho(\mathbf{x}'). \tag{3}$$

We refer to $\{\eta_\rho\}$ as the spectrum of RKHS, or the kernel eigenvalues. The number of nonzero kernel eigenvalues defines the dimension of the RKHS, which is infinite in the general setting. By the orthonormality of the functions $\phi_\rho(\mathbf{x})$ on $p(\mathbf{x})$, we can verify that the eigenfunction property is satisfied

$$T_K[\phi_\rho](\mathbf{x}') = \int p(\mathbf{x}) K(\mathbf{x}, \mathbf{x}') \phi_\rho(\mathbf{x}) d\mathbf{x} = \sum_{\rho'} \eta_{\rho'} \phi_{\rho'}(\mathbf{x}') \int p(\mathbf{x}) \phi_{\rho'}(\mathbf{x}) \phi_\rho(\mathbf{x}') d\mathbf{x} = \eta_\rho \phi_\rho(\mathbf{x}'). \tag{4}$$

As a consequence of the basis property of $\{\phi_\rho\}_{\rho=0}^{\infty}$, a square integrable function $f(\mathbf{x}) \in L_2(\mathcal{X}, p)$ can be expanded as:

$$f(\mathbf{x}) = \sum_{\rho=0}^{\infty} a_\rho \phi_\rho(\mathbf{x}) \ , \ a_\rho = \int p(\mathbf{x}) f(\mathbf{x}) \phi_\rho(\mathbf{x}) d\mathbf{x}. \tag{5}$$

By the reproducing property of the kernel, we find the identity $\langle \phi_\rho, \phi_\gamma \rangle_{\mathcal{H}} = \frac{\delta_{\rho,\gamma}}{\eta_\rho}$. Therefore the RKHS norm of a function f is

$$||f||_{\mathcal{H}}^2 = \langle f, f \rangle_{\mathcal{H}} = \sum_{\rho,\gamma} a_\rho a_\gamma \langle \phi_\rho, \phi_\gamma \rangle_{\mathcal{H}} = \sum_{\rho=0}^{\infty} \frac{a_\rho^2}{\eta_\rho}. \tag{6}$$

A function f is said to be a member of the RKHS $\mathcal{H}$ if and only if $||f||_{\mathcal{H}}^2 < \infty$.

Given a set of training examples $\mathcal{D} \equiv \{\mathbf{x}^\mu, y^\mu\}|_{\mu=1}^P$, the problem of interest is the minimization of the energy function $H[f;\mathcal{D}]$ with respect to functions $f \in \mathcal{H}$:

$$f^*(\mathbf{x}) = \text{argmin}_{f\in\mathcal{H}} H[f;\mathcal{D}], \qquad H[f;\mathcal{D}] \equiv \frac{1}{2\lambda}\sum_{\mu=1}^P \big(y^\mu - f(\mathbf{x}^\mu)\big)^2 + \frac{1}{2}\,\|f\|^2_{\mathcal{H}}\,. \tag{7}$$

Here, we explicitly denote dataset dependence $\mathcal{D}$. Labels y^μ are generated from a noisy target function:

$$y^\mu = \bar{f}(\mathbf{x}^\mu) + \epsilon^\mu, \quad \langle \epsilon^\mu, \epsilon^\nu\rangle = \sigma^2\delta^{\mu\nu}. \tag{8}$$

We define the null-space indices of the kernel as $\mathcal{N} = \{\rho|\eta_\rho = 0\}$ and its complement as $\mathcal{N}^\perp$. Since the learned function is always in the RKHS (the energy H includes a penalty on $||f||^2_{\mathcal{H}}$), it cannot place any power in the null modes indexed by $\mathcal{N}$ and therefore can only optimize over linear combinations of eigenfunctions indexed by $\mathcal{N}^\perp$. We introduce rescaled features $\psi_\rho = \sqrt{\eta_\rho}\phi_\rho$ for all $\rho \in \mathcal{N}^\perp$. The square integrable target function admits the decomposition

$$\bar{f}(\mathbf{x}) = \sum_{\rho\in\mathcal{N}^\perp} \overline{w}_\rho\psi_\rho(\mathbf{x}) + \sum_{\rho\in\mathcal{N}} a_\rho\phi_\rho(\mathbf{x}) = \overline{\mathbf{w}}\cdot\mathbf{\Psi}(\mathbf{x}) + \bar{\mathbf{a}}\cdot\mathbf{\Phi}(\mathbf{x}), \tag{9}$$

where we have introduced vectors $\overline{\mathbf{w}}, \bar{\mathbf{a}}, \mathbf{\Phi}, \mathbf{\Psi}$ of the appropriate dimensions. We stress that the assumption of a square integrable target $\overline{f}$ is identical to the condition that the target possess finite variance under the data density $p(\mathbf{x})$, a very mild assumption which gives our theory significant generality. The kernel regression task reduces to minimization of the energy function over the learned *weights* w_ρ:

$$\mathbf{w}^* = \text{argmin}_{\mathbf{w}} H(\mathbf{w};\mathcal{D}), \quad H(\mathbf{w};\mathcal{D}) \equiv \frac{1}{2\lambda}\sum_{\mu=1}^P \big(\mathbf{\Psi}(\mathbf{x}^\mu)\cdot(\bar{\mathbf{w}}-\mathbf{w}) + \bar{\mathbf{a}}\cdot\mathbf{\Phi}(\mathbf{x}^\mu) + \epsilon^\mu\big)^2 + \frac{1}{2}\|\mathbf{w}\|_2^2. \tag{10}$$

where $\bar{\mathbf{a}}$ contains the coefficients of the teacher for null modes indexed by $\mathcal{N}$. Generalization error and training error are defined as:

$$\begin{aligned} E_g(\mathbf{w}^*,\mathcal{D}) &\equiv \Big\langle \big(f^*(\mathbf{x}) - \bar{f}(\mathbf{x})\big)^2\Big\rangle_{\mathbf{x}} = (\mathbf{w}^*-\bar{\mathbf{w}})^\top\mathbf{\Lambda}(\mathbf{w}^*-\bar{\mathbf{w}}) + \|\bar{\mathbf{a}}\|^2 \\ E_{tr}(\mathbf{w}^*,\mathcal{D}) &\equiv \frac{1}{2P}\sum_{\mu=1}^P \big(\mathbf{\Psi}(\mathbf{x}^\mu)\cdot(\bar{\mathbf{w}}-\mathbf{w}^*) + \bar{\mathbf{a}}\cdot\mathbf{\Phi}(\mathbf{x}^\mu) + \epsilon^\mu\big)^2, \end{aligned} \tag{11}$$

where we introduced the diagonal matrix of the spectrum $\mathbf{\Lambda}_{\rho\gamma} \equiv \eta_\rho\delta_{\rho\gamma}$. The last term in $E_g(\mathcal{D})$, $||\bar{\mathbf{a}}||^2$, represents the inability of the kernel to place any power in modes indexed by $\mathcal{N}$ and is thus an irreducible estimation error, present even in the $P\to\infty$ limit, which we denote as $E_g(\infty)$. Our main goal is to average $E_g(\mathcal{D})$ and $E_{tr}(\mathcal{D})$ over all possible realizations of $\mathcal{D}$ of fixed size P.

Supplementary Note 2 - Replica Calculation

We perform replica calculation to find the expected value of training error, estimator and its RKHS norm as well as the generalization error. To set up our statistical mechanics problem, we first introduce the following partition function:

$$Z[J,K,\boldsymbol{\xi},\boldsymbol{\chi}] = \int d\mathbf{w} e^{-\beta H(\mathbf{w};\mathcal{D}) + J\frac{\beta P}{2}E_g(\mathbf{w},\mathcal{D}) + K\frac{\beta P}{2\lambda}E_{tr}(\mathbf{w},\mathcal{D}) + \beta\boldsymbol{\xi}\cdot\mathbf{w} + \frac{\beta}{2}\boldsymbol{\chi}^\top\mathbf{w}\mathbf{w}^\top\boldsymbol{\chi}}, \tag{12}$$

such that

$$E_g(\mathbf{w}^*, \mathcal{D}) = \lim_{\beta\to\infty} \frac{2}{\beta P}\frac{\partial}{\partial J}\log Z[J,K,\boldsymbol{\xi},\boldsymbol{\chi}]\Big|_{J,K,\boldsymbol{\xi},\boldsymbol{\chi}=0} + \|\bar{\mathbf{a}}\|^2$$
$$E_{tr}(\mathbf{w}^*, \mathcal{D}) = \lim_{\beta\to\infty} \frac{\lambda}{\beta P}\frac{\partial}{\partial K}\log Z[J,K,\boldsymbol{\xi},\boldsymbol{\chi}]\Big|_{J,K,\boldsymbol{\xi},\boldsymbol{\chi}=0} \tag{13}$$

since the integral in Supplementary Equation 12 concentrates around the weights $\mathbf{w}^*$ of the kernel regression solution in the $\beta \to \infty$ limit. Last two terms in the exponent of Supplementary Equation 12 is to calculate the expected estimator $f(\mathbf{x})$ and its correlation function via:

$$\sqrt{\eta_\alpha}\,\langle w_\alpha(\mathcal{D})\rangle_{\mathbf{w}} = \frac{1}{\beta}\frac{\partial}{\partial \xi'_\alpha}\log Z[J,K,\boldsymbol{\xi},\boldsymbol{\chi}]\Big|_{J,K,\boldsymbol{\xi},\boldsymbol{\chi}=0}$$
$$\sqrt{\eta_\alpha\eta_\beta}\,\langle w_\alpha(\mathcal{D})w_\beta(\mathcal{D})\rangle_{\mathbf{w}} = \frac{1}{\beta}\frac{\partial^2}{\partial \chi'_\alpha\partial\chi'_\beta}\log Z[J,K,\boldsymbol{\xi},\boldsymbol{\chi}]\Big|_{J,K,\boldsymbol{\xi},\boldsymbol{\chi}=0}, \tag{14}$$

where the primed quantities are defined as $\boldsymbol{\xi} = \boldsymbol{\Lambda}^{1/2}\boldsymbol{\xi}'$ and $\boldsymbol{\chi} = \boldsymbol{\Lambda}^{1/2}\boldsymbol{\chi}'$. Hence one can read out:

$$\langle f(\mathbf{x},\mathcal{D})\rangle_{\mathbf{w}} = \sum_\alpha \sqrt{\eta_\alpha}\,\langle w_\alpha(\mathcal{D})\rangle_{\mathbf{w}}\,\phi_\alpha(\mathbf{x})$$
$$\langle f(\mathbf{x},\mathcal{D})f(\mathbf{x}',\mathcal{D})\rangle_{\mathbf{w}} = \sum_{\alpha\beta} \sqrt{\eta_\alpha\eta_\beta}\,\langle w_\alpha(\mathcal{D})w_\beta(\mathcal{D})\rangle_{\mathbf{w}}\,\phi_\alpha(\mathbf{x})\phi_\beta(\mathbf{x}') \tag{15}$$

Here the $\langle .. \rangle_{\mathbf{w}}$ refers to ensemble average over all possible $\mathbf{w}$'s with probability weights given by $e^{-\beta H(\mathbf{w};\mathcal{D})}$. We will eventually consider the $\beta \to \infty$ limit to obtain the quantities above corresponding to the kernel regression solution.

In order to perform the training set average $\langle \mathcal{O}(\mathcal{D})\rangle_{\mathcal{D}}$ for the quantities above, we must average $\log Z$ over all possible training samples and noises. Resorting to the replica trick, averaging $\log Z$ reduces to averaging n-times replicated partition function Z^n:

$$\langle \log Z\rangle_{\mathcal{D}} = \lim_{n\to 0}\frac{\langle Z^n\rangle_{\mathcal{D}} - 1}{n}$$
$$\langle Z^n\rangle_{\mathcal{D}} = e^{-\frac{n\beta}{2}(\bar{\mathbf{W}}-\boldsymbol{\xi})^\top\bar{\mathbf{w}}}\int\left(\prod_{a=1}^n d\mathbf{w}^a\right)e^{-\frac{\beta}{2}\sum_{a=1}^n \mathbf{w}^{a\top}(\mathbf{I}-JP\boldsymbol{\Lambda}-\boldsymbol{\chi}\boldsymbol{\chi}^\top)\mathbf{w}^a-\beta\bar{\mathbf{W}}^\top\sum_{a=1}^n\mathbf{w}^a}$$
$$\times\left\langle e^{-\frac{\beta(1-K)}{2\lambda}\sum_{a=1}^n\left(\mathbf{w}^a\cdot\boldsymbol{\Psi}(\mathbf{x})+\bar{\mathbf{a}}\cdot\boldsymbol{\Phi}(\mathbf{x})+\epsilon^a\right)^2}\right\rangle_{\mathbf{x},\{\epsilon^a\}}^P, \tag{16}$$

where we shifted $\mathbf{w}^a \to \mathbf{w}^a + \bar{\mathbf{w}}$ and defined $\bar{\mathbf{W}} = (\bar{\mathbf{w}} - \boldsymbol{\xi}) - (\boldsymbol{\chi}^\top\bar{\mathbf{w}})\boldsymbol{\chi}$ for notational simplicity. Now we can average over quenched disorder introduced due to the training samples and noise before integrating out the thermal degrees of freedom.

Averaging over Quenched Disorder

The quantity of interest is the following:

$$\left\langle e^{-\frac{\beta(1-K)}{2\lambda}\sum_{a=1}^n\left(\mathbf{w}^a\cdot\boldsymbol{\Psi}(\mathbf{x})+\bar{\mathbf{a}}\cdot\boldsymbol{\Phi}(\mathbf{x})+\epsilon^a\right)^2}\right\rangle_{\mathbf{x},\{\epsilon^a\}}. \tag{17}$$

Rather than integrating over $\mathbf{x}$, we integrate over $q^a = \mathbf{w}^a \cdot \mathbf{\Psi}(\mathbf{x}) + \bar{\mathbf{a}} \cdot \mathbf{\Phi}(\mathbf{x}) + \epsilon^a$, which is itself a random variable with mean and covariance:

$$\begin{aligned}
\boldsymbol{\mu}^a &\equiv \langle q^a \rangle = \langle \mathbf{w}^a \cdot \mathbf{\Psi}(\mathbf{x}) \rangle + \left\langle \bar{\mathbf{a}} \cdot \mathbf{\Phi}(\mathbf{x}) \right\rangle + \langle \epsilon^a \rangle = \sqrt{\eta_0} w_0^a, \\
\mathbf{C}^{ab} &\equiv \langle q^a q^b \rangle = \mathbf{w}^{a\top} \langle \mathbf{\Psi}(\mathbf{x}) \mathbf{\Psi}(\mathbf{x})^\top \rangle \mathbf{w}^b + \bar{\mathbf{a}}^\top \left\langle \mathbf{\Phi}(\mathbf{x}) \mathbf{\Phi}(\mathbf{x})^\top \right\rangle \bar{\mathbf{a}} + \langle \epsilon^a \epsilon^b \rangle \\
&= \mathbf{w}^{a\top} \mathbf{\Lambda} \mathbf{w}^b + \mathbf{\Sigma}^{ab},
\end{aligned} \tag{18}$$

where $\mathbf{\Sigma} = \left(\|\bar{\mathbf{a}}\|^2 + \sigma^2\right)\mathbf{1}\mathbf{1}^\top$ is the covariance matrix of noise across replicas. Computation of this mean and covariance relied on the orthogonality of the eigenfunctions. Notice that the target modes on the null space of the kernel act as noise on training labels, quantifying the effect of out-of-RKHS target functions. From here on, we take $\|\bar{\mathbf{a}}\|^2 = 0$ for simplicity. At the end of the calculation it can be recovered by shifting $\sigma^2 \to \sigma^2 + \|\bar{\mathbf{a}}\|^2$ and $E_g \to E_g + \|\bar{\mathbf{a}}\|^2$. Note that the noise-free part of the diagonal elements represents the generalization error in a single replica i.e. $\mathbf{C}^{aa} = E_g^a + \sigma^2$, while off-diagonal elements give the overlap of the weights across different replicas. In the limit $\beta \to \infty$, we expect these two quantities to be equal as the optimal weights averaged over training samples across different replicas will be the same due to the convexity of the problem.

First, we omit the zero mode μ by considering only kernels with zero constant terms (such that $\eta_0 = 0$), however it is straightforward to include it in the rest of the calculation and we give the contribution from zero mode in our final expression. Next, by observing that q^a is a summation of many uncorrelated random variables ($\left\langle \psi_\rho(\mathbf{x}) \psi_{\rho'}(\mathbf{x}) \right\rangle_{\mathbf{x}\sim p(\mathbf{x})} = \eta_\rho \delta_{\rho\rho'}$) and a Gaussian noise, we approximate the probability distribution of q^a by a multivariate Gaussian with its mean and covariance given by Supplementary Equation 18:

$$P(\{q^a\}) = \frac{1}{\sqrt{(2\pi)^n \det(\mathbf{C})}} \exp\Bigg(-\frac{1}{2} \sum_{a,b} q^a \left(\mathbf{C}^{ab}\right)^{-1} q^b \Bigg). \tag{19}$$

This approximation is further validated with the excellent match of our theory to simulations. Then the average over quenched disorder reduces to:

$$\begin{aligned}
\left\langle e^{-\frac{\beta(1-K)}{2\lambda} \sum_{a=1}^n \left((\mathbf{w}^a - \bar{\mathbf{w}})\cdot \mathbf{\Psi}(\mathbf{x}) + \epsilon^a\right)^2} \right\rangle_{\mathbf{x},\{\epsilon^a\}} &\approx \int \{dq^a\} P(\{q^a\}) \exp\Bigg(-\frac{\beta_K}{2\lambda} \sum_{a=1}^n (q^a)^2 \Bigg) \\
&= \exp\Bigg(-\frac{1}{2} \log\det\Bigg(\mathbf{I} + \frac{\beta_K}{\lambda}\mathbf{C}\Bigg)\Bigg),
\end{aligned} \tag{20}$$

where we defined $\beta_K \equiv \beta(1-K)$ for notational convenience. Combining everything together, the dataset averaged replicated partition function becomes:

$$\begin{aligned}
\langle Z^n \rangle_{\mathcal{D}} = e^{-\frac{n\beta}{2}(\bar{\mathbf{W}} - \boldsymbol{\xi})^\top \bar{\mathbf{w}}} \int \Bigg(\prod_{a=1}^n d\mathbf{w}^a \Bigg) e^{-\frac{\beta}{2} \sum_{a=1}^n \mathbf{w}^{a\top} (\mathbf{I} - JP\mathbf{\Lambda} - \boldsymbol{\chi}\boldsymbol{\chi}^\top) \mathbf{w}^a - \beta \bar{\mathbf{W}}^\top \sum_{a=1}^n \mathbf{w}^a} \\
\times e^{-\frac{P}{2} \log\det\left(\mathbf{I} + \frac{\beta_K}{\lambda}\mathbf{C}\right)}
\end{aligned} \tag{21}$$

Using the definitions Supplementary Equation 18, we insert conjugate variables through the following identity:

$$1 = \left(\frac{iP}{2\pi}\right)^{\frac{n(n+1)}{2}} \int \Bigg(\prod_{a\geq b} d\mathbf{C}^{ab} d\hat{\mathbf{C}}^{ab} \Bigg) \exp\Bigg[-P \sum_{a\geq b} \hat{\mathbf{C}}^{ab} \Big(\mathbf{C}^{ab} - \mathbf{w}^{a\top} \mathbf{\Lambda} \mathbf{w}^b - \mathbf{\Sigma}^{ab} \Big) \Bigg] \tag{22}$$

Here, integral over $\hat{\mathbf{C}}$ runs over the imaginary axis and we explicitly scaled it by P. Then defining:

$$G_E = \frac{1}{2}\log\det\left(\mathbf{I} + \frac{\beta_K}{\lambda}\mathbf{C}\right) \tag{23}$$

$$e^{-G_S} = \int\left(\prod_{a=1}^{n} d\mathbf{w}^a\right)\exp\left(-\frac{\beta}{2}\sum_{a\geq b}\mathbf{w}^{a\top}\left((\mathbf{I} - JP\boldsymbol{\Lambda} - \boldsymbol{\chi}\boldsymbol{\chi}^\top)\mathbf{I}^{ab} - \frac{2P}{\beta}\boldsymbol{\Lambda}\hat{\mathbf{C}}^{ab}\right)\mathbf{w}^b - \beta\bar{\mathbf{W}}^\top\sum_{a=1}^{n}\mathbf{w}^a\right),$$

we obtain:

$$\langle Z^n\rangle = e^{\frac{n(n+1)}{2}\log\left(\frac{iP}{2\pi}\right) - \frac{n\beta}{2}(\bar{\mathbf{W}}-\boldsymbol{\xi})^\top\bar{\mathbf{w}}}\int\left(\prod_{a\geq b} d\mathbf{C}^{ab}d\hat{\mathbf{C}}^{ab}\right)\exp\left[-P\sum_{a\geq b}\hat{\mathbf{C}}^{ab}(\mathbf{C}^{ab} - \boldsymbol{\Sigma}^{ab}) - PG_E - G_S\right]. \tag{24}$$

Therefore, we only need to evaluate the integral in G_S. First we would like to express the ordered sum $\sum_{a\geq b}\mathbf{w}^{a\top}\left((\mathbf{I} - JP\boldsymbol{\Lambda} - \boldsymbol{\chi}\boldsymbol{\chi}^\top)\mathbf{I}^{ab} - \frac{2P}{\beta}\boldsymbol{\Lambda}\hat{\mathbf{C}}^{ab}\right)\mathbf{w}^b$ as an unordered sum over a, b. Note that

$$\sum_{a,b}\mathbf{w}^{a\top}\left((\mathbf{I} - JP\boldsymbol{\Lambda} - \boldsymbol{\chi}\boldsymbol{\chi}^\top)\mathbf{I}^{ab} - \frac{2P}{\beta}\boldsymbol{\Lambda}\hat{\mathbf{C}}^{ab}\right)\mathbf{w}^b$$
$$= 2\sum_{a\geq b}\mathbf{w}^{a\top}\left((\mathbf{I} - JP\boldsymbol{\Lambda} - \boldsymbol{\chi}\boldsymbol{\chi}^\top)\mathbf{I}^{ab} - \frac{2P}{\beta}\boldsymbol{\Lambda}\hat{\mathbf{C}}^{ab}\right)\mathbf{w}^b - \sum_{a,b}\mathbf{w}^{a\top}\left((\mathbf{I} - JP\boldsymbol{\Lambda} - \boldsymbol{\chi}\boldsymbol{\chi}^\top)\mathbf{I}^{ab} - \frac{2P}{\beta}\boldsymbol{\Lambda}\mathrm{diag}(\hat{\mathbf{C}})^{ab}\right)\mathbf{w}^b$$

Hence, we obtain:

$$\sum_{a\geq b}\mathbf{w}^{a\top}\left((\mathbf{I} - JP\boldsymbol{\Lambda} - \boldsymbol{\chi}\boldsymbol{\chi}^\top)\mathbf{I}^{ab} - \frac{2P}{\beta}\boldsymbol{\Lambda}\hat{\mathbf{C}}^{ab}\right)\mathbf{w}^b = \sum_{a,b}\mathbf{w}^{a\top}\mathbf{X}^{ab}\mathbf{w}^b,$$

where we defined:

$$\mathbf{X}^{ab} = \left(\mathbf{I} - JP\boldsymbol{\Lambda} - \boldsymbol{\chi}\boldsymbol{\chi}^\top\right)\mathbf{I}^{ab} - \frac{P}{\beta}\boldsymbol{\Lambda}\left(\hat{\mathbf{C}} + \mathrm{diag}(\hat{\mathbf{C}})\right)^{ab}. \tag{25}$$

In order to evaluate the Gaussian integral, we will cast the function and target weights into an nM dimensional vector:

$$\mathbf{w} = \begin{pmatrix}\mathbf{w}^1 & \mathbf{w}^2 & .. & \mathbf{w}^a & .. & \mathbf{w}^n\end{pmatrix}_{nM\times 1}$$
$$\bar{\mathbf{W}}_{\otimes n} = \begin{pmatrix}\bar{\mathbf{W}} & \bar{\mathbf{W}} & \dots & \bar{\mathbf{W}}\end{pmatrix}_{nM\times 1} \tag{26}$$

Furthermore, we introduce the $nM \times nM$ matrix $\mathbf{X}$ as:

$$\mathbf{X} = \begin{pmatrix}\mathbf{X}^{11} & \mathbf{X}^{12} & \dots & \dots & \mathbf{X}^{1n}\\ \mathbf{X}^{21} & \mathbf{X}^{22} & \dots & \dots & \mathbf{X}^{2n}\\ \vdots & \vdots & \ddots & & \vdots\\ \vdots & \dots & \mathbf{X}^{ab} & \ddots & \vdots\\ \mathbf{X}^{n1} & \dots & \dots & \dots & \mathbf{X}^{nn}\end{pmatrix}_{nM\times nM} \tag{27}$$

Finally we denote the integration measure as $\mathcal{D}\mathbf{w} = \prod_{a,\rho} dw_\rho^a$. With these definitions, G_S becomes:

$$e^{-G_S} = \int \mathcal{D}\mathbf{w}\, e^{-\frac{\beta}{2}\mathbf{w}^\top \mathbf{X}\mathbf{w} - \beta \bar{\mathbf{W}}_{\otimes n}^\top \mathbf{w}} \tag{28}$$

Hence, we turned the integral in G_S to a simple Gaussian integral. The result is:

$$e^{-G_S} = \left(\frac{2\pi}{\beta}\right)^{\frac{nM}{2}} \big(\det \mathbf{X}\big)^{-\frac{1}{2}} \exp\left(\frac{\beta}{2}\bar{\mathbf{W}}_{\otimes n}^\top \mathbf{X}^{-1}\bar{\mathbf{W}}_{\otimes n}\right). \tag{29}$$

Now the integral in Supplementary Equation 24 can be evaluated using the method of steepest descent. In Supplementary Equation 24, we see that all the terms in the exponent is $\mathcal{O}(n)$. Furthermore, we will use P as the saddle point parameter going to infinity with a proper scaling. Therefore, defining the following function:

$$\begin{aligned} S[\mathbf{C}, \hat{\mathbf{C}}, \boldsymbol{\mu}, \hat{\boldsymbol{\mu}}] &= \frac{1}{n}\sum_{a\geq b} \hat{\mathbf{C}}^{ab}(\mathbf{C}^{ab} - \boldsymbol{\Sigma}^{ab}) + \frac{1}{nP}\left(PG_E + G_S + \frac{n\beta}{2}(\bar{\mathbf{W}} - \boldsymbol{\xi})^\top \bar{\mathbf{w}}\right) \\ G_E &= \frac{1}{2}\log\det\left(\mathbf{I} + \frac{\beta_K}{\lambda}\mathbf{C}\right) \\ G_S &= \frac{1}{2}\log\det \mathbf{X} - \frac{\beta}{2}\bar{\mathbf{W}}_{\otimes n}^\top \mathbf{X}^{-1}\bar{\mathbf{W}}_{\otimes n}, \end{aligned} \tag{30}$$

we obtain:

$$\begin{aligned} \langle \log Z\rangle &= \lim_{n\to 0}\frac{1}{n}\big(\langle Z^n\rangle - 1\big), \\ \langle Z^n\rangle &= e^{\frac{n(n+1)}{2}\log\left(\frac{iP}{2\pi}\right) + \frac{nM}{2}\log\frac{2\pi}{\beta}} \int \left(\prod_{a\geq b} d\mathbf{C}^{ab} d\hat{\mathbf{C}}^{ab}\right) e^{-nPS[\mathbf{C},\hat{\mathbf{C}}]}. \end{aligned} \tag{31}$$

The reader may question the dependence of various quantities in S on P, since we are taking a $P \to \infty$ limit. This is because we want to keep our treatment general. Depending on the kernel and data distribution, there are other quantities here that can scale with P. Specific examples will be given.

Replica Symmetry and Saddle Point Equations

In order to proceed with the saddle point integration, we further assume replica symmetry relying on the convexity of the problem:

$$\begin{aligned} C^0 &= \mathbf{C}^{aa}, & \hat{C}^0 &= \hat{\mathbf{C}}^{aa}, \\ C &= \mathbf{C}^{a\neq b}, & \hat{C} &= \hat{\mathbf{C}}^{a\neq b}. \end{aligned} \tag{32}$$

Therefore, we have $\mathbf{C} = (C_0 - C)\mathbf{I} + C\mathbf{1}\mathbf{1}^\top$ and $\hat{\mathbf{C}} = (\hat{C}_0 - \hat{C})\mathbf{I} + \hat{C}\mathbf{1}\mathbf{1}^\top$. In this case, the matrix $\mathbf{X}$ has the form:

$$\mathbf{X} = \begin{pmatrix} \mathbf{X}_0 & \mathbf{X}_1 & \mathbf{X}_1 & \dots & \mathbf{X}_1 \\ \mathbf{X}_1 & \mathbf{X}_0 & \mathbf{X}_1 & \dots & \mathbf{X}_1 \\ \mathbf{X}_1 & \mathbf{X}_1 & \mathbf{X}_0 & \dots & \vdots \\ \vdots & \dots & \dots & \ddots & \vdots \\ \mathbf{X}_1 & \dots & \dots & \dots & \mathbf{X}_0 \end{pmatrix} = \mathbf{I}_{n\times n} \otimes (\mathbf{X}_0 - \mathbf{X}_1)_{M\times M} + \mathbf{1}_{n\times n} \otimes (\mathbf{X}_1)_{M\times M}, \tag{33}$$

where:

$$\mathbf{X}_0 \equiv \mathbf{X}^{aa} = \big(\mathbf{I} - JP\boldsymbol{\Lambda} - \boldsymbol{\chi}\boldsymbol{\chi}^\top\big) - \frac{2P\hat{C}_0}{\beta}\boldsymbol{\Lambda}$$
$$\mathbf{X}_1 \equiv \mathbf{X}^{a\neq b} = -\frac{P\hat{C}}{\beta}\boldsymbol{\Lambda}.$$

It is straightforward to calculate the inverse of this matrix using Sherman-Morrison-Woodbury formula $(A+B)^{-1} = A^{-1} - A^{-1}BA^{-1}(I+BA^{-1})^{-1}$:

$$\begin{aligned}\mathbf{X}^{-1} &= \mathbf{I}_n \otimes (\mathbf{X}_0 - \mathbf{X}_1)^{-1} - \big(\mathbf{1}_n \otimes (\mathbf{X}_0 - \mathbf{X}_1)^{-1}\mathbf{X}_1(\mathbf{X}_0 - \mathbf{X}_1)^{-1}\big)\big(\mathbf{I}_n \otimes \mathbf{I}_M + \mathbf{1}_n \otimes \mathbf{X}_1(\mathbf{X}_0 - \mathbf{X}_1)^{-1}\big)^{-1} \\ &= \mathbf{I}_n \otimes \mathbf{G}^{-1} - \mathbf{1}_n \otimes \mathbf{G}^{-1}\mathbf{X}_1\mathbf{G}^{-1} + \mathcal{O}(n),\end{aligned}$$

where we defined,

$$\mathbf{G} \equiv \mathbf{X}_0 - \mathbf{X}_1 = \mathbf{I} - \frac{P(2\hat{C}_0 - \hat{C}) + J\beta P}{\beta}\boldsymbol{\Lambda} - \boldsymbol{\chi}\boldsymbol{\chi}^\top, \tag{34}$$

for shorthand notation. Hence, we get:

$$\bar{\mathbf{W}}_{\otimes n}^\top \mathbf{X}^{-1}\bar{\mathbf{W}}_{\otimes n} = n\bar{\mathbf{W}}^\top\mathbf{G}^{-1}\bar{\mathbf{W}} + \mathcal{O}(n^2). \tag{35}$$

We also need to calculate the determinant of this matrix which can be done by using Gaussian elimination method to bring it into a block-triangular form. The result is:

$$\det\mathbf{X} = \det(\mathbf{X}_0 - \mathbf{X}_1)^{n-1}\det\big(\mathbf{X}_0 + (n-1)\mathbf{X}_1\big) = \det(\mathbf{X}_0 - \mathbf{X}_1)^{n-1}\det(\mathbf{X}_0 - \mathbf{X}_1 + n\mathbf{X}_1). \tag{36}$$

Taylor expanding the last term using $\det(\mathbf{I} + n\mathbf{C}) = 1 + n\operatorname{Tr}\mathbf{C} + \mathcal{O}(n^2)$, we obtain:

$$\log\det\mathbf{X} = n\log\det\mathbf{G} + n\operatorname{Tr}\Big(\mathbf{X}_1\mathbf{G}^{-1}\Big) + \mathcal{O}(n^2) = n\log\det\mathbf{G} - n\frac{P\hat{C}}{\beta}\operatorname{Tr}\boldsymbol{\Lambda}\mathbf{G}^{-1} + \mathcal{O}(n^2). \tag{37}$$

Next, using the matrix determinant lemma $\det\Big(A + uv^T\Big) = \det(A)(1 + v^TA^{-1}u)$, we obtain:

$$\begin{aligned}\det\left(\mathbf{I} + \frac{\beta_K}{\lambda}\mathbf{C}\right) &= \big[1 + \frac{\beta_K}{\lambda}(C_0 - C)\big]^n\left(1 + n\frac{\beta_K C}{\lambda + \beta_K(C_0 - C)}\right), \\ \Rightarrow \log\det\left(\mathbf{I} + \frac{\beta_K}{\lambda}\mathbf{C}\right) &= n\log\left(1 + \frac{\beta(1-K)}{\lambda}(C_0 - C)\right) + n\frac{\beta(1-K)C}{\lambda + \beta(1-K)(C_0 - C)},\end{aligned} \tag{38}$$

where we recovered $\beta_K = \beta(1-K)$. Finally, we need to simplify $\sum_{a\geq b}\hat{\mathbf{C}}^{ab}(\mathbf{C}^{ab} - \boldsymbol{\Sigma}^{ab})$ under the replica symmetry up to leading order in n:

$$\sum_{a\geq b}\hat{\mathbf{C}}^{ab}(\mathbf{C}^{ab} - \boldsymbol{\Sigma}^{ab}) = n\big(\hat{C}_0(C_0 - \sigma^2) - \frac{1}{2}\hat{C}(C - \sigma^2)\big). \tag{39}$$

Therefore, under replica symmetry, the function S given in Supplementary Equation 30 simplifies to:

$$\begin{aligned}S[\mathbf{C}, \hat{\mathbf{C}}] =& \hat{C}_0(C_0 - \sigma^2) - \frac{1}{2}\hat{C}(C - \sigma^2) + \frac{1}{2}\log\left(1 + \frac{\beta(1-K)}{\lambda}(C_0 - C)\right) + \frac{1}{2}\frac{\beta(1-K)C}{\lambda + \beta(1-K)(C_0 - C)} \\ &+ \frac{1}{2P}\left(\log\det\mathbf{G} - \frac{P\hat{C}}{\beta}\operatorname{Tr}\boldsymbol{\Lambda}\mathbf{G}^{-1}\right) - \frac{\beta}{2P}\bar{\mathbf{W}}^\top\mathbf{G}^{-1}\bar{\mathbf{W}} + \frac{\beta}{2P}(\bar{\mathbf{W}} - \boldsymbol{\xi})^\top\bar{\mathbf{w}},\end{aligned} \tag{40}$$

where we recall that $\mathbf{G} = \mathbf{I} - \frac{P(2\hat{C}_0 - \hat{C}) + J\beta P}{\beta}\boldsymbol{\Lambda} - \boldsymbol{\chi}\boldsymbol{\chi}^\top$. The saddle point equations of S with respect to C_0 and C are simple:

$$\frac{\partial S}{\partial C} = 0 \Rightarrow \boxed{\hat{C} = \frac{\beta^2(1-K)^2 C}{\big(\lambda + \beta(1-K)(C_0 - C)\big)^2}},$$
$$\frac{\partial S}{\partial C_0} = 0 \Rightarrow \boxed{\hat{C}_0 = \frac{1}{2}\hat{C} - \frac{1}{2}\frac{\beta(1-K)}{\lambda + \beta(1-K)(C_0 - C)}}. \tag{41}$$

The equation $\partial S/\partial \hat{C} = 0$ yields:

$$C = \frac{P\hat{C}}{\beta^2}\operatorname{Tr}\boldsymbol{\Lambda}\mathbf{G}^{-1}\boldsymbol{\Lambda}\mathbf{G}^{-1} + \bar{\mathbf{W}}^\top\mathbf{G}^{-1}\boldsymbol{\Lambda}\mathbf{G}^{-1}\bar{\mathbf{W}} + \sigma^2, \tag{42}$$

and the equation $\partial S/\partial \hat{C}_0 = 0$ yields:

$$C_0 = C + \frac{1}{\beta}\operatorname{Tr}\boldsymbol{\Lambda}\mathbf{G}^{-1}. \tag{43}$$

Two commonly appearing forms are:

$$\begin{aligned} \kappa \equiv \lambda + \beta(1-K)(C_0 - C) &= \lambda + (1-K)\operatorname{Tr}\boldsymbol{\Lambda}\mathbf{G}^{-1}, \\ \frac{2\hat{C}_0 - \hat{C}}{\beta} = -\frac{(1-K)}{\lambda + \beta(1-K)(C_0 - C)} &= -\frac{(1-K)}{\kappa}. \end{aligned} \tag{44}$$

Plugging second equation to the expression for $\mathbf{G}$, we get:

$$\mathbf{G} = \mathbf{I} + \frac{P}{\kappa}(1 - K - J\kappa)\boldsymbol{\Lambda} - \boldsymbol{\chi}\boldsymbol{\chi}^\top, \tag{45}$$

hence we obtain the following implicit equation:

$$\kappa = \lambda + (1-K)\operatorname{Tr}\boldsymbol{\Lambda}\left(\mathbf{I} + \frac{P}{\kappa}(1 - K - J\kappa)\boldsymbol{\Lambda} - \boldsymbol{\chi}\boldsymbol{\chi}^\top\right)^{-1}. \tag{46}$$

In terms of κ, final saddle point equations reduce to:

$$\begin{aligned} \hat{C}_0^* &= \frac{1}{2}\hat{C}^* - \frac{1}{2}\frac{\beta(1-K)}{\kappa}, \\ \hat{C}^* &= \frac{\beta^2(1-K)^2 C^*}{\kappa^2}, \\ C_0^* &= C^* + \frac{\kappa - \lambda}{(1-K)\beta}, \\ C^* &= C^*(1-K)^2\frac{P}{\kappa^2}\operatorname{Tr}\boldsymbol{\Lambda}\mathbf{G}^{-1}\boldsymbol{\Lambda}\mathbf{G}^{-1} + \bar{\mathbf{W}}^\top\mathbf{G}^{-1}\boldsymbol{\Lambda}\mathbf{G}^{-1}\bar{\mathbf{W}} + \sigma^2. \end{aligned} \tag{47}$$

Here * indicates the quantities that give the saddle point. Finally, solving for C^* in the last equation, we obtain:

$$C^* = \frac{1}{1 - (1-K)^2\frac{P}{\kappa^2}\operatorname{Tr}\boldsymbol{\Lambda}\mathbf{G}^{-1}\boldsymbol{\Lambda}\mathbf{G}^{-1}}\left(\bar{\mathbf{W}}^\top\mathbf{G}^{-1}\boldsymbol{\Lambda}\mathbf{G}^{-1}\bar{\mathbf{W}} + \sigma^2\right). \tag{48}$$

Having obtained the saddle points, we can evaluate the saddle point integral. In the limit $P \to \infty$, the dominant contribution is:

$$\langle Z^n \rangle \approx e^{-nPS[\mathbf{C}^*, \hat{\mathbf{C}}^*]}. \tag{49}$$

Taking the $n \to 0$ limit and plugging in the saddle point solutions to the expression Supplementary Equation 40, we obtain the free energy $\langle \log Z \rangle = -PS$ to be:

$$\begin{aligned}
\langle \log Z \rangle &= \frac{P}{2}\frac{\kappa - \lambda}{\kappa} - \frac{P}{2}\log\frac{\kappa}{\lambda} - \frac{P}{2}\log\det\mathbf{G} - \frac{\beta P(1-K)}{2}\frac{\sigma^2}{\kappa} + \frac{\beta}{2}\bar{\mathbf{W}}^\top \mathbf{G}^{-1}\bar{\mathbf{W}} - \frac{\beta}{2}(\bar{\mathbf{W}} - \boldsymbol{\xi})^\top \bar{\mathbf{w}}, \\
\kappa &= \lambda + (1-K)\,\mathrm{Tr}\,\boldsymbol{\Lambda}\mathbf{G}^{-1}, \\
\mathbf{G} &= \mathbf{I} + \frac{P}{\kappa}(1 - K - J\kappa)\boldsymbol{\Lambda} - \boldsymbol{\chi}\boldsymbol{\chi}^\top, \\
\bar{\mathbf{W}} &= (\bar{\mathbf{w}} - \boldsymbol{\xi}) - (\boldsymbol{\chi}^\top \bar{\mathbf{w}})\boldsymbol{\chi}.
\end{aligned} \tag{50}$$

Generalization Error

Now, we can calculate $E_g = \lim_{\beta\to\infty} \frac{2}{\beta P}\frac{\partial}{\partial J}\langle \log Z \rangle |_{J,K,\boldsymbol{\xi},\boldsymbol{\chi}=0}$. Recall that κ is itself a function of J. Explicit calculation and $\beta \to \infty$ limit yields:

$$E_g = \Big(\partial_J \kappa(0) + \kappa^2(0)\Big)\sum_{\rho=1}^{N} \frac{\eta_\rho \bar{w}_\rho^2}{\big(\kappa(0) + P\eta_\rho\big)^2} + \sigma^2 \frac{\partial_J \kappa(0)}{\kappa^2(0)}, \tag{51}$$

where

$$\begin{aligned}
\frac{\partial_J \kappa(0)}{\kappa^2(0)} &= \frac{1}{1 - \sum_\rho \frac{P\eta_\rho^2}{(\kappa + P\eta_\rho)^2}} \sum_\rho \frac{P\eta_\rho^2}{(\kappa + P\eta_\rho)^2} \equiv \frac{\gamma}{1-\gamma}, \\
\kappa \equiv \kappa(0) &= \lambda + \sum_\rho \frac{\kappa\eta_\rho}{P\eta_\rho + \kappa},
\end{aligned} \tag{52}$$

where we defined $\gamma = \sum_\rho \frac{P\eta_\rho^2}{(\kappa + P\eta_\rho)^2}$. In terms of these quantities, averaged generalization error becomes:

$$\boxed{E_g = \frac{1}{1-\gamma}\sum_{\rho=1}^{N} \frac{\eta_\rho}{\big(\kappa + P\eta_\rho\big)^2}\big(\kappa^2 \bar{w}_\rho^2 + (\sigma^2 + \|\bar{\mathbf{a}}\|^2)P\eta_\rho\big) + \|\bar{\mathbf{a}}\|^2 + \frac{1+\gamma}{1-\gamma}\kappa^2 \frac{\eta_0 \bar{w}_0^2}{(\kappa + 2P\eta_0)^2}}. \tag{53}$$

For completeness, we recovered $\|\bar{\mathbf{a}}\|^2 \neq 0$ (Supplementary Note 1) to account for the case where target function $\bar{f}(\mathbf{x}) = \bar{\mathbf{w}} \cdot \boldsymbol{\Psi}(\mathbf{x}) + \sum_{\rho \in \mathcal{N}} a_\rho \Phi_\rho(\mathbf{x})$ has components out-of-RKHS. The last term accounts for the contribution to E_g from the zero mode when $\eta_0 \neq 0$. Note that at this point we have already taken the $P \to \infty$ limit. P still appears in these expressions to consider different scaling limits for kernel eigenvalues.

This expression generalizes Eq. (4) in main text to cases where the target function has modes not expressible by the kernel. These modes contribute an irreducible component to the generalization error, as well as act as noise on expressible modes. Based on this expression, we can define another effective noise $\tilde{\sigma} = \sigma^2 + \|\bar{\mathbf{a}}\|^2$, which can lead to non-monotonic learning curves even in the absence of label noise ($\sigma = 0$). An example of this phenomenon is provided in Supplementary Figure 2.

Training Error

Next, we calculate the expected training error defined as:

$$E_{tr} = \lim_{\beta\to\infty} \frac{\lambda}{\beta P} \frac{\partial}{\partial K} \left\langle \log Z \right\rangle \Big|_{J,K,\boldsymbol{\xi},\boldsymbol{\chi}=0}. \tag{54}$$

First we shall calculate:

$$\partial_K \kappa(0) = -\frac{\kappa^2 \delta}{1-\gamma}, \quad \kappa = \lambda + \kappa \sum_\rho \frac{\eta_\rho}{\kappa + P\eta_\rho}, \quad \gamma = \sum_\rho \frac{P\eta_\rho^2}{(\kappa + P\eta_\rho)^2}, \quad \delta = \sum_\rho \frac{\eta_\rho}{(\kappa + P\eta_\rho)^2} \tag{55}$$

A cumbersome calculation yields:

$$\frac{\partial}{\partial K} \left\langle \log Z \right\rangle\big|_{J,K,\boldsymbol{\xi}=0} = -\frac{P\beta}{2}\left(\frac{\kappa\delta + \gamma - 1}{\kappa}\right)\big(E_g(P) + \sigma^2\big) + \frac{P}{2}(\kappa\delta + \gamma), \tag{56}$$

where E_g is defined above Supplementary Equation 53. By using the summation forms of κ, γ and δ, one can show that:

$$\kappa\delta + \gamma = 1 - \frac{\lambda}{\kappa}. \tag{57}$$

Plugging this back in, we obtain:

$$\frac{\partial}{\partial K} \left\langle \log Z \right\rangle\big|_{J,K,\boldsymbol{\xi}=0} = \frac{P\beta}{2}\frac{\lambda}{\kappa^2}\big(E_g(P) + \sigma^2\big) + \frac{P}{2}\left(1 - \frac{\lambda}{\kappa}\right). \tag{58}$$

Hence average case training error becomes:

$$E_{tr}(P) = \lim_{\beta\to\infty} \frac{\lambda}{\beta P}\frac{\partial}{\partial K} \left\langle \log Z \right\rangle\big|_{J,K,\boldsymbol{\xi},\boldsymbol{\chi}=0} = \frac{\lambda^2}{\kappa^2}\big(E_g(P) + \sigma^2\big). \tag{59}$$

This is consistent with the expectation that in the $\beta \to \infty$ limit, kernel machine can fit all training samples perfectly when $\lambda = 0$. We also note that the same relation between training and generalization errors were also obtained in other works [4, 5] using random matrix theory.

Expected Estimator and the Correlation Function

Finally, we calculate the RKHS weights of the expected function and its variance:

$$\begin{aligned} \sqrt{\eta_\alpha}\left\langle w_\alpha^* \right\rangle_{\mathcal{D}} &= \lim_{\beta\to\infty} \frac{1}{\beta}\frac{\partial}{\partial \xi'_\alpha} \left\langle \log Z \right\rangle\Big|_{J,K,\boldsymbol{\xi},\boldsymbol{\chi}=0} \\ \sqrt{\eta_\alpha \eta_\beta}\left\langle w_\alpha^* w_\beta^* \right\rangle_{\mathcal{D}} &= \lim_{\beta\to\infty} \frac{1}{\beta}\frac{\partial^2}{\partial \chi'_\alpha \partial \chi'_\beta} \left\langle \log Z \right\rangle\Big|_{J,K,\boldsymbol{\xi},\boldsymbol{\chi}=0}, \end{aligned} \tag{60}$$

where the derivatives are with respect to $\xi'_\alpha = \xi_\alpha/\sqrt{\eta_\alpha}$ and $\chi'_\alpha = \chi_\alpha/\sqrt{\eta_\alpha}$, respectively. Taking derivatives for each entry of $\boldsymbol{\xi}'$, we obtain:

$$\frac{1}{\beta}\frac{\partial}{\partial \xi'_\alpha} \left\langle \log Z \right\rangle = \sqrt{\eta_\alpha}\bar{w}_\alpha - \frac{\kappa\sqrt{\eta_\alpha}(\bar{w}_\alpha - \xi_\alpha)}{P\eta_\alpha + \kappa} = \frac{P\eta_\alpha\sqrt{\eta_\alpha}\bar{w}_\alpha + \kappa\sqrt{\eta_\alpha}\xi_\alpha}{P\eta_\alpha + \kappa}. \tag{61}$$

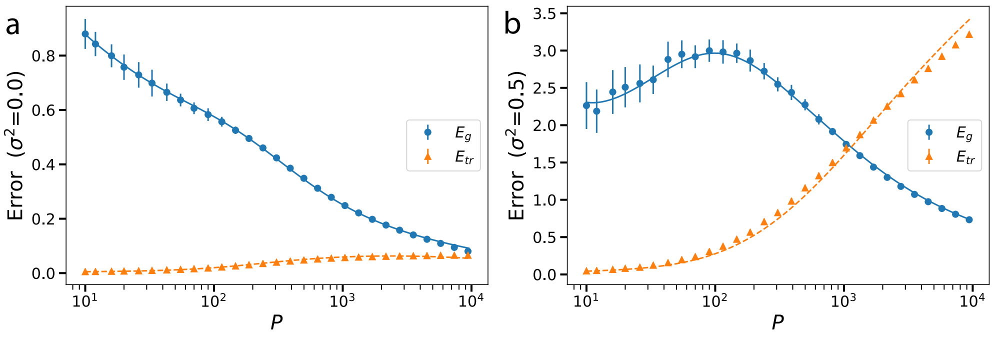

Supplementary Figure 1: Kernel regression with Gaussian RBF $K(\mathbf{x}, \mathbf{x}') = e^{-\frac{1}{2D\omega^2}||x-x'||^2}$ for MNIST with kernel bandwidth $\omega = 0.1$, ridge parameter $\lambda = 0.01$ and noise variance $\sigma^2 = 0, 0.5$. Theoretical predictions for E_g (solid blue line) and E_{tr} (dashed orange line) are in excellent agreement with experiment (blue dots and orange triangles, respectively). Error bars represent standard deviation over 30 trials. **a.** When no label noise is present, E_{tr} is always smaller than E_g. **b.** When noise is non-zero, E_{tr} may exceed E_g due to explicit presence of label noise in the loss function.

Hence the *average estimator* has the following form:

$$\langle f^*(\mathbf{x}; P)\rangle_{\mathcal{D}} = \sum_\rho \frac{P\eta_\rho}{P\eta_\rho + \kappa} \bar{w}_\rho \psi_\rho(\mathbf{x}), \tag{62}$$

which approaches to the target function as $P \to \infty$. Note that the learned function can only express the components which span the RKHS. If the target function has out-of-RKHS components, those will never be learned.

A related result was given in [6] which used a similar technique to calculate the expected estimator. With our notation, their perturbative result for uniform spherical datasets and dot product kernels reads (Eq. (24) in [6]):

$$\langle f^*(\mathbf{x}; P)\rangle_{\mathcal{D}} = \sum_\rho \left(\frac{P\eta_\rho}{P\eta_\rho + \lambda} - \frac{P\eta_\rho}{(P\eta_\rho + \lambda)^2} \lambda \sum_\rho \frac{\eta_\rho}{P\eta_\rho + \lambda} + \dots \right) \bar{w}_\rho \psi_\rho(\mathbf{x}), \tag{63}$$

where λ is the ridge parameter and $\dots$ represent the higher order corrections in their perturbative setting. We obtain the same result result if we expand our expression for $\langle f^*(\mathbf{x}; P)\rangle_{\mathcal{D}}$ in a power series of $\epsilon = (\kappa - \lambda)$ assuming ϵ is small, and use $\kappa - \lambda = \kappa \sum_\rho \frac{\eta_\rho}{P\eta_\rho + \kappa}$:

$$\frac{P\eta_\rho}{P\eta_\rho + \kappa} = \frac{P\eta_\rho}{P\eta_\rho + \lambda} - \frac{P\eta_\rho}{(P\eta_\rho + \lambda)^2} \lambda \sum_\rho \frac{\eta_\rho}{P\eta_\rho + \lambda} + \dots \tag{64}$$

Hence, our result includes corrections not captured in [6].

Finally, we want to calculate the correlation function of the estimator. Given the partition function $Z = \int d\mathbf{w} e^{-\beta H(\mathbf{w};\mathcal{D}) + \beta \boldsymbol{\xi}\cdot\mathbf{w} + \frac{\beta}{2}\boldsymbol{\chi}^\top \mathbf{w}\mathbf{w}^\top \boldsymbol{\chi}}$, notice that the variance:

$$\frac{1}{\beta^2} \frac{\partial^2}{\partial\xi_\alpha \partial\xi_\beta} \langle \log Z \rangle = \left\langle \langle w_\alpha w_\beta \rangle_{\mathbf{w}} - \langle w_\alpha \rangle_{\mathbf{w}} \langle w_\beta \rangle_{\mathbf{w}} \right\rangle_{\mathcal{D}} = \frac{1}{\beta} \frac{\kappa}{P\eta_\alpha + \kappa} \delta_{\alpha\beta} \tag{65}$$

vanishes as $\beta \to \infty$ since there is a unique solution. However, there is variance to the estimator due to averaging over different training sets which is given by:

$$\langle w_\alpha w_\beta \rangle_{\mathbf{w},\mathcal{D}} - \langle w_\alpha \rangle_{\mathbf{w},\mathcal{D}} \langle w_\beta \rangle_{\mathbf{w},\mathcal{D}}, \tag{66}$$

and it is finite as $\beta \to \infty$. The first term, the eigenfunction expansion coefficients of the correlation function of the estimator $\langle f(\mathbf{x}) f(\mathbf{x}')\rangle$, can be calculated by taking two derivatives of $\langle \log Z\rangle$ with respect to $\boldsymbol{\chi}'$. To simplify the calculation, we first redefine $\mathbf{G} \equiv \left(\mathbf{I} + \frac{P}{\kappa} - \boldsymbol{\Lambda}^{1/2}\boldsymbol{\chi}'\boldsymbol{\chi}'^{\top}\boldsymbol{\Lambda}^{1/2}\right)^{-1}$ by setting $J = K = 0$ and introduce the notation $\partial_\alpha \equiv \frac{\partial}{\partial \chi'_\alpha}$ for notational simplicity. First, we calculate the derivatives of κ:

$$\begin{aligned}
\partial_\alpha \kappa &= -\operatorname{Tr} \boldsymbol{\Lambda}\mathbf{G}^{-1}\left(-\frac{P}{\kappa^2}\boldsymbol{\Lambda}\partial_\alpha\kappa - \boldsymbol{\Lambda}^{1/2}\partial_\alpha(\boldsymbol{\chi}'\boldsymbol{\chi}'^{\top})\boldsymbol{\Lambda}^{1/2}\right)\mathbf{G}^{-1} \\
&= \partial_\alpha\kappa \frac{P}{\kappa^2}\operatorname{Tr}\boldsymbol{\Lambda}^2\mathbf{G}^{-2} + \operatorname{Tr}\boldsymbol{\Lambda}\mathbf{G}^{-1}\boldsymbol{\Lambda}^{1/2}\partial_\alpha(\boldsymbol{\chi}'\boldsymbol{\chi}'^{\top})\boldsymbol{\Lambda}^{1/2}\mathbf{G}^{-1} \\
&= \partial_\alpha\kappa \frac{P}{\kappa^2}\operatorname{Tr}\boldsymbol{\Lambda}^2\mathbf{G}^{-2} + 2\sum_\rho \eta_\rho \mathbf{G}^{-1}_{\rho\alpha}\sqrt{\eta_\alpha}\left(\sum_\sigma \sqrt{\eta_\sigma}\chi'_\sigma \mathbf{G}^{-1}_{\sigma\rho}\right).
\end{aligned} \tag{67}$$

Hence, we find that:

$$\partial_{\boldsymbol{\chi}'}\kappa|_{\boldsymbol{\chi}'=0} = \partial_{\boldsymbol{\chi}'}\mathbf{G}|_{\boldsymbol{\chi}'=0} = 0. \tag{68}$$

This greatly simplifies the second derivative of κ:

$$\begin{aligned}
\partial_\alpha\partial_\beta\kappa|_{\boldsymbol{\chi}'=0} &= \left[\partial_\alpha\partial_\beta\kappa\frac{P}{\kappa^2}\operatorname{Tr}\boldsymbol{\Lambda}^2\mathbf{G}^{-2} + 2\sum_\rho \eta_\rho\mathbf{G}^{-1}_{\rho\alpha}\sqrt{\eta_\alpha\eta_\beta}\mathbf{G}^{-1}_{\beta\rho}\right]\Bigg|_{\boldsymbol{\chi}'=0} \\
&= (\partial_\alpha\partial_\beta\kappa|_{\boldsymbol{\chi}'=0})\sum_\rho \frac{P\eta_\rho^2}{(P\eta_\rho+\kappa)^2} + \frac{2\kappa^2}{P}\frac{P\eta_\alpha^2}{(P\eta_\alpha+\kappa)^2}\delta_{\alpha\beta} \\
&= \frac{2\kappa^2}{P}\frac{1}{1-\gamma}\frac{P\eta_\alpha^2}{(P\eta_\alpha+\kappa)^2}\delta_{\alpha\beta},
\end{aligned} \tag{69}$$

where $\gamma = \sum_\rho \frac{P\eta_\rho^2}{(P\eta_\rho+\kappa)^2}$ as defined before. Now we calculate the variance of the expected function:

$$\begin{aligned}
\sqrt{\eta_\alpha\eta_\beta}\,\langle w^*_\alpha w^*_\beta\rangle_{\mathcal{D}} &= \lim_{\beta\to\infty}\frac{1}{\beta}\partial_\alpha\partial_\beta\,\langle\log Z\rangle\,\big|_{\boldsymbol{\xi}',\boldsymbol{\chi}'=0} \\
&= \frac{P}{2}\frac{\sigma^2}{\kappa^2}\partial_\alpha\partial_\beta\kappa + \sqrt{\eta_\alpha\eta_\beta}\bar{w}_\alpha\bar{w}_\beta - \kappa\frac{\sqrt{\eta_\alpha\eta_\beta}\bar{w}_\alpha\bar{w}_\beta}{P\eta_\beta+\kappa} - \kappa\frac{\sqrt{\eta_\alpha\eta_\beta}\bar{w}_\alpha\bar{w}_\beta}{P\eta_\alpha+\kappa} \\
&\quad - \frac{1}{2}\bar{\mathbf{W}}^\top\mathbf{G}^{-1}\left(-\frac{P}{\kappa^2}\boldsymbol{\Lambda}\partial_\alpha\partial_\beta\kappa - \boldsymbol{\Lambda}^{1/2}\partial_\alpha\partial_\beta(\boldsymbol{\chi}'\boldsymbol{\chi}'^\top)\boldsymbol{\Lambda}^{1/2}\right)\mathbf{G}^{-1}\bar{\mathbf{W}} \\
&= \frac{1}{1-\gamma}\left(\sigma^2 + \kappa^2\sum_\rho\frac{\eta_\rho\bar{w}_\rho^2}{(P\eta_\rho+\kappa)^2}\right)\frac{P\eta_\alpha^2}{(P\eta_\alpha+\kappa)^2}\delta_{\alpha\beta} + \kappa^2\frac{\eta_\alpha\bar{w}_\alpha^2}{(P\eta_\alpha+\kappa)^2}\delta_{\alpha\beta} \\
&\quad + \frac{P\eta_\beta\sqrt{\eta_\alpha\eta_\beta}\bar{w}_\alpha\bar{w}_\beta}{P\eta_\beta+\kappa} - \kappa\frac{\sqrt{\eta_\alpha\eta_\beta}\bar{w}_\alpha\bar{w}_\beta}{P\eta_\alpha+\kappa}.
\end{aligned} \tag{70}$$

Now we can calculate the coefficients of covariance of the estimator:

$$\sqrt{\eta_\alpha\eta_\beta}\,\langle w^*_\alpha w^*_\beta\rangle_{\mathcal{D}} - \sqrt{\eta_\alpha\eta_\beta}\,\langle w^*_\alpha\rangle_{\mathcal{D}}\,\langle w^*_\beta\rangle_{\mathcal{D}} \tag{71}$$
$$= \frac{1}{1-\gamma}\left(\sigma^2 + \kappa^2\sum_\rho \frac{\eta_\rho \bar{w}_\rho^2}{(P\eta_\rho+\kappa)^2}\right)\frac{P\eta_\alpha^2}{(P\eta_\alpha+\kappa)^2}\delta_{\alpha\beta} + \kappa^2\frac{\eta_\alpha\bar{w}_\alpha^2}{(P\eta_\alpha+\kappa)^2}\delta_{\alpha\beta}$$
$$+ \frac{(P\eta_\alpha+\kappa)P\eta_\beta\sqrt{\eta_\alpha\eta_\beta}\bar{w}_\alpha\bar{w}_\beta}{(P\eta_\alpha+\kappa)(P\eta_\beta+\kappa)} - \kappa\frac{(P\eta_\beta+\kappa)\sqrt{\eta_\alpha\eta_\beta}\bar{w}_\alpha\bar{w}_\beta}{(P\eta_\alpha+\kappa)(P\eta_\beta+\kappa)} - \frac{P^2\eta_\alpha\eta_\beta\sqrt{\eta_\alpha\eta_\beta}\bar{w}_\alpha\bar{w}_\beta}{(P\eta_\alpha+\kappa)(P\eta_\beta+\kappa)}$$
$$= \boxed{\frac{1}{1-\gamma}\left(\sigma^2 + \kappa^2\sum_\rho \frac{\eta_\rho \bar{w}_\rho^2}{(P\eta_\rho+\kappa)^2}\right)\frac{P\eta_\alpha^2}{(P\eta_\alpha+\kappa)^2}\delta_{\alpha\beta} + \kappa^2\frac{\sqrt{\eta_\alpha\eta_\beta}\bar{w}_\alpha\bar{w}_\beta}{(P\eta_\alpha+\kappa)(P\eta_\beta+\kappa)}(\delta_{\alpha\beta}-1)}.$$

Hence the covariance of the estimator is:

$$Cov[\langle f^*(\mathbf{x};P)f^*(\mathbf{x}';P)\rangle_{\mathcal{D}}] = \sum_{\alpha\beta}\left(\langle w^*_\alpha w^*_\beta\rangle_{\mathcal{D}} - \langle w^*_\alpha\rangle_{\mathcal{D}}\langle w^*_\beta\rangle_{\mathcal{D}}\right)\psi_\alpha(\mathbf{x})\psi_\beta(\mathbf{x}'). \tag{72}$$

Note that the generalization error can be decomposed as:

$$\langle E_g\rangle_{\mathcal{D}} = \int d\mathbf{x}\,\langle f^{*2}(\mathbf{x})\rangle_{\mathcal{D}} - 2\int d\mathbf{x}\,\langle f^*(\mathbf{x})\rangle_{\mathcal{D}}\,\bar{f}(\mathbf{x}) + \int d\mathbf{x}\bar{f}(\mathbf{x})^2 \tag{73}$$

From the calculation above, we can find:

$$\int d\mathbf{x}\,\langle f^{*2}(\mathbf{x})\rangle_{\mathcal{D}} - \int d\mathbf{x}\,\langle f^*(\mathbf{x})\rangle_{\mathcal{D}}\,\langle f^*(\mathbf{x})\rangle_{\mathcal{D}} = \frac{\gamma}{1-\gamma}\left(\sigma^2+\kappa^2\sum_\rho\frac{\eta_\rho\bar{w}_\rho^2}{(P\eta_\rho+\kappa)^2}\right)$$
$$\int d\mathbf{x}\,\langle f^*(\mathbf{x})\rangle_{\mathcal{D}}\,\langle f^*(\mathbf{x})\rangle_{\mathcal{D}} = \sum_\rho\frac{P^2\eta_\rho^3\bar{w}_\rho^2}{(P\eta_\rho+\kappa)^2}$$
$$\int d\mathbf{x}\,\langle f^*(\mathbf{x})\rangle_{\mathcal{D}}\,\bar{f}(\mathbf{x}) = \sum_\rho\frac{P\eta_\rho^2\bar{w}_\rho^2}{P\eta_\rho+\kappa}$$
$$\int d\mathbf{x}\bar{f}(\mathbf{x})^2 = \sum_\rho\eta_\rho\bar{w}_\rho^2, \tag{74}$$

where the first line is the contribution to generalization error due to estimator variance. Hence generalization error is:

$$\langle E_g\rangle_{\mathcal{D}} = \frac{\gamma}{1-\gamma}\left(\sigma^2+\kappa^2\sum_\rho\frac{\eta_\rho\bar{w}_\rho^2}{(P\eta_\rho+\kappa)^2}\right) + \sum_\rho\frac{P^2\eta_\rho^3\bar{w}_\rho^2}{(P\eta_\rho+\kappa)^2} - 2\sum_\rho\frac{P\eta_\rho^2\bar{w}_\rho^2}{P\eta_\rho+\kappa} + \sum_\rho\eta_\rho\bar{w}_\rho^2$$
$$= \frac{\gamma}{1-\gamma}\sigma^2 + \frac{\kappa^2}{1-\gamma}\sum_\rho\frac{\eta_\rho\bar{w}_\rho^2}{(P\eta_\rho+\kappa)^2}, \tag{75}$$

which is what we obtained before. In terms of the variance of the estimator, E_g is also equal to:

$$\langle E_g\rangle_{\mathcal{D}} = \underbrace{\frac{\gamma}{1-\gamma}\left(\sigma^2+\kappa^2\sum_\rho\frac{\eta_\rho\bar{w}_\rho^2}{(P\eta_\rho+\kappa)^2}\right)}_{\textbf{variance } V} + \underbrace{\kappa^2\sum_\rho\frac{\eta_\rho\bar{w}_\rho^2}{(P\eta_\rho+\kappa)^2}}_{\textbf{bias } B}. \tag{76}$$

This is the bias-variance decomposition of generalization error in our setting where the bias term is monotonically decreasing while the variance term is solely responsible for any non-monotonicity appearing in the generalization error.

Supplementary Note 3 - White Band-limited RKHS Spectrum

As a simple but illuminating example, we consider a kernel with band-limited spectrum: $\eta_\rho = 0$ for $\rho > N$. For simplicity, we study the case where the spectrum is white $\eta_\rho = \frac{1}{N}$ for all $\rho = 1, ..., N$ and study this system in the large N, large P limit with $\alpha = P/N \sim \mathcal{O}(1)$. We normalize the target power in the first N modes $\sum_{\rho=1}^{N} \overline{w}_\rho^2 = N$. Furthermore, the coefficients for the target function are a_ρ for all $\rho > N$: $f^*(\mathbf{x}) = \sum_{\rho=1}^{N} \overline{w}_\rho \psi_\rho(\mathbf{x}) + \sum_{\rho=N+1}^{\infty} a_\rho \phi_\rho(\mathbf{x})$. At the saddle point, the implicit equation κ can be solved explicitly in the $P, N \to \infty$ limit with $\alpha = P/N \sim \mathcal{O}(1)$.

$$\kappa_\lambda(\alpha) = \frac{1}{2}\left[(\lambda + 1 - \alpha) + \sqrt{(\lambda + 1 - \alpha)^2 + 4\lambda\alpha}\right], \tag{77}$$

The generalization error Supplementary Equation 53 becomes:

$$E_g = \frac{\kappa_\lambda(\alpha)^2 + \tilde{\sigma}^2\alpha}{(\kappa_\lambda(\alpha) + \alpha)^2 - \alpha} + E_g(\infty) \tag{78}$$

where $E_g(\infty) = \sum_{\rho > N} a_\rho^2$ is the asymptotic value of the generalization error and $\tilde{\sigma}^2 = \sigma^2 + E_g(\infty)$ is the effective noise. The first term is the noiseless contribution to E_g while second term is only due to the noise in target coming from both the explicit noise σ^2 and the variance of modes in the null-space of the kernel $E_g(\infty)$. In the following we will simply denote $\tilde{\sigma}^2$ with σ^2 since it is only relevant noise appearing in the generalization error formula. The generalization error asymptotically falls faster in the absence of noise:

$$\begin{aligned} E_g - E_g(\infty) &\sim \frac{\sigma^2}{\alpha},\ \alpha \to \infty,\ (\sigma > 0), \\ E_g - E_g(\infty) &\sim \frac{\lambda^2}{\alpha^2},\ \alpha \to \infty,\ (\sigma = 0). \end{aligned} \tag{79}$$

Furthermore, explicit calculation reveals that the noiseless term monotonically decreases with α, while the noise term has a maximum at $\alpha = 1 + \lambda$ and its maximum is given by:

$$\left.\frac{\gamma}{1-\gamma}\right|_{\alpha = 1+\lambda} = \frac{1}{2\sqrt{\lambda}} \frac{1}{\sqrt{\lambda} + \sqrt{\lambda + 1}} \tag{80}$$

In the presence of noise, generalization error diverges when $\lambda \to 0$, while finite λ smoothes out the learning curve. In machine learning, this non-monotonic behavior of generalization error is called "double-descent", and signals overfitting of the noise in the data [7, 8, 9]. Diverging generalization error further implies a first order phase transition when $\alpha = 1 + \lambda = 1$. This can be seen by examining the first derivative of the free energy Supplementary Equation 50 in $\beta \to \infty$ limit:

$$\frac{1}{\beta}\frac{\partial S}{\partial \alpha} = \frac{\sigma^2}{2\kappa}\frac{\alpha\gamma}{1-\gamma} \sim \frac{\sigma^2}{2\lambda}\theta(\alpha - 1) + \mathcal{O}(\lambda)\ ,\ \lambda \to 0, \tag{81}$$

where the approximation is valid for $\lambda \ll 1$. We observe that, in the absence of noise, there is no phase transition while in the noisy case, there is a sharp discontinuity and divergence when $\lambda = 0$. Although there is no phase transition in the strict sense of a non-analytic free energy except for the case $\lambda = 0$, we describe whether there is non-monotonicity or not as separate phases of the kernel machine.

We would like to understand what combinations of (λ, σ^2) leads to non-monotonicity in generalization error. One can obtain the exact phase boundary for non-monotonicity by studying the zeros

of $\partial E_g/\partial\alpha$ given by:

$$\frac{\partial E_g(\alpha)}{\partial\alpha} = -\frac{1}{2} + \frac{(\alpha+\lambda-5)(\alpha+\lambda+1)^2 + 2(\lambda+2)(3\alpha+1+\lambda) - 2\sigma^2(\alpha-1-\lambda)}{2\left((\alpha+1+\lambda)^2-4\alpha\right)^{3/2}} = 0 \tag{82}$$

Explicit calculation yields:

$$\sigma^2_{\text{critical}} > \begin{cases} g(\lambda) & \lambda < 1 \\ 2\lambda+1 & \lambda \geq 1 \end{cases}, \tag{83}$$

where $g(\lambda)$ is:

$$g(\lambda) = 3\lambda(3\lambda+2-2\sqrt{1+\lambda}\sqrt{9\lambda+1}\cos\theta),$$
$$\theta = \frac{1}{3}\left(\pi + \tan^{-1}\frac{8\sqrt{\lambda}}{9\lambda(3\lambda+2)-1}\right). \tag{84}$$

In the non-monotonic region, we further observe that the curve $\sigma^2_{\text{critical}} = 2\lambda+1$ for $\lambda < 1$ separates two regions with a single and double local extrema. Above this curve, there is a single local maximum corresponding to a peak while below there is a local minimum followed by a local maximum (double-descent). As a side note, we emphasize that double-descent might occur when target functions have out-of-RKHS components even without label corruption. In Supplementary Figure 2, the target function is chosen such that $E_g(\infty) = 0.2$ in Supplementary Equation 79. Since this causes non-zero effective noise, we observe double-descent even in the absence of label noise.

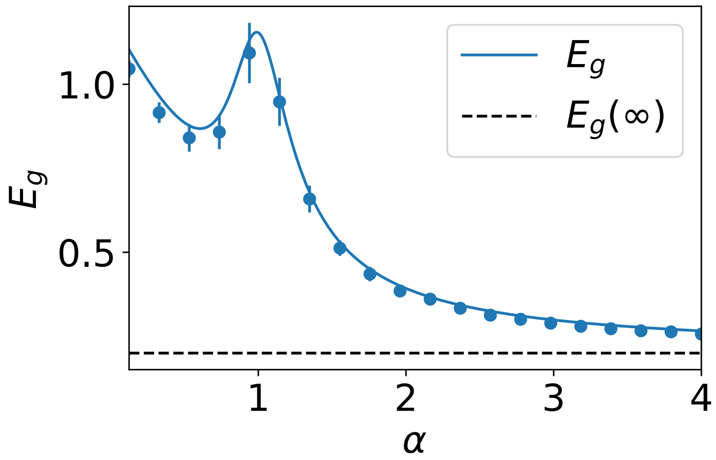

Supplementary Figure 2: Zero noise target function with components outside of the RKHS. We simulated the linear kernel $K(\mathbf{x},\mathbf{x}') = \sum_{\rho=1}^{N}\eta_\rho x_\rho x'_\rho$ where $\mathbf{x}\sim\mathcal{N}(0,\mathbf{I})$ are $N = 960$ dimensional uncorrelated Gaussian inputs and $\eta_\rho = 0$ for $\rho > 800$ and $\eta_\rho = 1$, otherwise. Target is a linear function $\bar{f}(\mathbf{x}) = \boldsymbol{\beta}^\top\mathbf{x}$ with $\boldsymbol{\beta}\sim\mathcal{N}(0,\frac{1}{N}\mathbf{I})$. This experiment demonstrates that out-of-RKHS components generate an irreducible error $E_g(\infty)$ (here $E_g(\infty) = 0.2$) and acts as effective noise, producing a double descent peak. The ridge parameter is chosen to be $\lambda = 0.01$. Error bars represent the standard deviation over 50 trials.

Although large λ regularizes the learning and avoids an overfitting peak, too large λ will also slow down the learning as can be seen from the asymptotic limit of Supplementary Equation 82 in λ:

$$\frac{\partial E_g(\alpha)}{\partial\alpha} = -\frac{2}{\lambda} + \frac{3(2\alpha+1)+\sigma}{\lambda^2} + \mathcal{O}\left(\frac{1}{\lambda^3}\right) \tag{85}$$

To find an optimal choice of ridge parameter, we study the first derivative of E_g with respect to λ and find that there is an optimal λ for a given noise level σ^2 independent of α:

$$\frac{\partial E_g(\alpha)}{\partial \lambda} = \frac{2\alpha(\lambda - \sigma^2)}{\left((\alpha+1+\lambda)^2 - 4\alpha\right)^{3/2}} = 0, \quad \Rightarrow \quad \lambda^* = \sigma^2 \tag{86}$$

This simple relation holds for all α and also indicates that the optimal choice of regularization leads to a monotonic learning curve as expected (See Fig. 3 in the main text). Note that the error due to the noise term is decreasing, while the noise-independent term is increasing with λ.

Finally, we numerically plot the α at which learning curve peak occurs as a function of noise with varying λ levels in Supplementary Figure 3. We observe that for large noise levels, location of the peak gets closer to $\alpha = 1 + \lambda$.

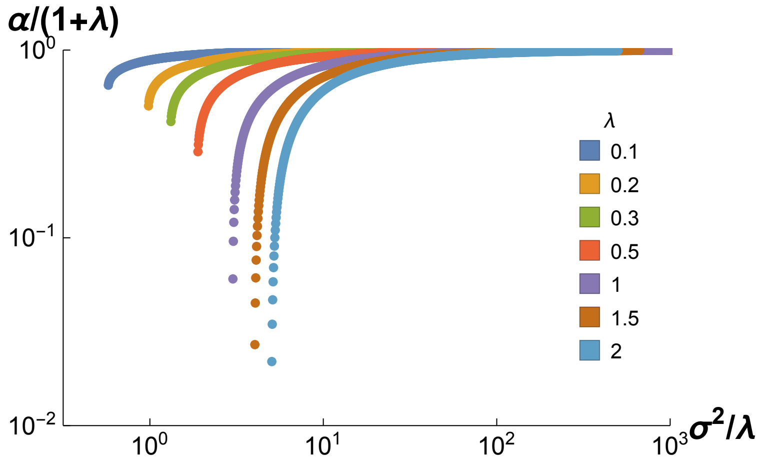

Supplementary Figure 3: Numerical calculation of $\alpha/(1+\lambda)$ as a function of σ^2/λ for varying ridge parameter λ.

Connection to Random Matrices

The quantities of interest in the generalization formula for the white band-limited spectrum are related to the distribution of random Wishart Matrices. In particular, let $\mathbf{X} \in \mathbb{R}^{N\times P}$ be a random Gaussian matrix with entries $X_{ij} \sim \mathcal{N}(0,1)$, then the quantity $\kappa_\lambda(\alpha)$ can be directly related to the following Stieltjes transform (resolvent) of the matrix $\frac{1}{N}\mathbf{X}\mathbf{X}^\top + \lambda\mathbf{I}$. The relationship is

$$\kappa_\lambda(\alpha) = \lambda + \frac{\lambda}{N}\mathrm{Tr}\left\langle \left(\frac{1}{N}\mathbf{X}\mathbf{X}^\top + \lambda\mathbf{I}\right)^{-1}\right\rangle_{\{X_{ij}\}} = \frac{1}{2}\left[(1+\lambda-\alpha) + \sqrt{(1+\lambda-\alpha)^2 + 4\lambda\alpha}\right], \tag{87}$$

where $\alpha = P/N$ in the $P, N \to \infty$ limit [10, 11]. If one wanted to compute the average case (over design matrices $\mathbf{X}$) generalization error for linear regression, this random matrix arises naturally from the solution to the regularized least-squares regression loss

$$\min_{\mathbf{w}} ||\frac{1}{\sqrt{N}}\mathbf{X}^\top\mathbf{w} - \mathbf{y}||^2 + \lambda||\mathbf{w}||^2 \implies \mathbf{w}^* = \frac{1}{\sqrt{N}}\left(\frac{1}{N}\mathbf{X}\mathbf{X}^\top + \lambda\mathbf{I}\right)^{-1}\mathbf{X}\mathbf{y}. \tag{88}$$

If the target values are produced by a teacher $\mathbf{y} = \frac{1}{\sqrt{N}}\mathbf{X}^\top\overline{\mathbf{w}}$ then

$$E_g = \overline{\mathbf{w}}^\top\left\langle\left(\frac{1}{\lambda}\mathbf{X}\mathbf{X}^\top + N\mathbf{I}\right)^{-2}\right\rangle\overline{\mathbf{w}} = -\frac{\partial}{\partial N}\overline{\mathbf{w}}^\top\left\langle\left(\frac{1}{\lambda}\mathbf{X}\mathbf{X}^\top + N\mathbf{I}\right)^{-1}\right\rangle\overline{\mathbf{w}}. \tag{89}$$

This matrix, and its average over the design $\mathbf{X}$, is also studied intensively in previous work on the generalization error of kernel regression [12, 13, 14]. Differentiation in N of this average matrix gives the desired result in the $N \to \infty$ limit

$$E_g = \frac{\kappa_\lambda(\alpha)^2}{(\kappa_\lambda(\alpha)+\alpha)^2 - \alpha} \lim_{N\to\infty} \left[||\overline{\mathbf{w}}||^2/N \right] \tag{90}$$

Since the distribution of each $\mathbf{x}^\mu$ is isotropic, all covariance eigenvalues are equal and each component w_ρ is learned at an identical rate. This expression is the same as our expression for the white band limited case, Supplementary Equation 79.

Next, we apply our findings to rotation invariant kernels and find that generalization error decomposes into different learning episodes which are individually described by the same formula we derived here in a special setting.

Supplementary Note 4 - Rotation Invariant Kernels

Here, we consider a widely used class of kernels left invariant under the rotations of the inputs: $K(\mathbf{Ox}, \mathbf{Ox}') = K(\mathbf{x}, \mathbf{x}'))$. We start by decomposing rotation invariant kernels into their spherical and radial directions:

Lemma 1. *Let $\mathcal{F}_\mathbf{r}$ be the set of functions that are invariant to all rotations that leave the vector $\mathbf{r} \in \mathbb{S}^{D-1}$ unchanged for all $f \in \mathcal{F}_\mathbf{r}$ and all orthogonal matrices $\mathbf{O} \in \mathbb{R}^{D\times D}$ with $\mathbf{Or} = \mathbf{r}$, $f(\mathbf{Ox}) = f(\mathbf{x})$. Any function $f \in \mathcal{F}_\mathbf{r}$ admits a decomposition*

$$f(\mathbf{x}) = \sum_k a_k(||\mathbf{x}||) Q_k^{(D-1)}(\hat{\mathbf{x}} \cdot \mathbf{r}), \tag{91}$$

where $Q_k^{(D-1)}(z)$ are the Gegenbauer polynomials (see Supplementary Note 6 for review).

Proof. For f to be invariant under the set of rotations which leave the vector $\mathbf{r}$ invariant, the restriction of f to spherical shells of radius $||\mathbf{x}|| = R$ must also be invariant under rotations. For fixed radius R, the set of all functions that are rotation invariant lie in span$\{Q_k(\mathbf{r}^\top \cdot /||\cdot||)\}$, since the Gegenbauer polynomials are complete with respect to the measure of inner products on $\mathbb{S}^{D-1}$. Repeating this decomposition for each restriction radius $||\mathbf{x}||$ gives radial dependent coefficients $a_k(||\mathbf{x}||)$. □

Using this lemma, we have the following decomposition for rotation invariant kernels ($K(\mathbf{Ox}, \mathbf{Ox}') = K(\mathbf{x}, \mathbf{x}')$) by first considering the rotation $\mathbf{O}$'s that leave $\mathbf{x}$ unchanged and then by considering the rotation $\mathbf{O}$'s that leave $\mathbf{x}'$ unchanged:

$$K(\mathbf{x}, \mathbf{x}') = \sum_k g_k(||\mathbf{x}||, ||\mathbf{x}'||) Q_k(\mathbf{x} \cdot \mathbf{x}'). \tag{92}$$

To calculate the eigenspectrum, we insert an ansatz of the form $\phi_{zkm}(\mathbf{x}) = R_{z,k}(||\mathbf{x}||) Y_{km}(\hat{\mathbf{x}})$ to the eigenvalue problem

$$\begin{aligned}
&\int d\mathbf{x} K(\mathbf{x}, \mathbf{x}') p(\mathbf{x}) \phi_{zkm}(\mathbf{x}) \\
&\quad = \int_0^\infty d||\mathbf{x}|| p(||\mathbf{x}||) \sum_{k'm'} g_{k'}(||\mathbf{x}|, ||\mathbf{x}'||) R_{z,k}(||\mathbf{x}||) Y_{k'm'}(\hat{\mathbf{x}}') \int_{\mathbb{S}^{D-1}} d\hat{\mathbf{x}}\, Y_{km}(\hat{\mathbf{x}}) Y_{k'm'}(\hat{\mathbf{x}}) \\
&\quad = Y_{km}(\hat{\mathbf{x}}') \int_0^\infty d||\mathbf{x}|| p(||\mathbf{x}||) g_k(||\mathbf{x}|, ||\mathbf{x}'||) R_{z,k}(||\mathbf{x}||) = \eta_{z,k} R_{z,k}(||\mathbf{x}'||) Y_{km}(\mathbf{x}'),
\end{aligned} \tag{93}$$

which gives a collection of radial eigenvalue problems (one for each degree k of spherical harmonics)

$$\int_0^\infty d||\mathbf{x}||p(||\mathbf{x}||)g_k(||\mathbf{x}||,||\mathbf{x}'||)R_{z,k}(||\mathbf{x}||) = \eta_{z,k}R_{z,k}(||\mathbf{x}'||). \tag{94}$$

For each, k, we solve the integral eigenvalue problem for a set of functions $\{R_{z,k}(||\mathbf{x}||)\}_z$ that are orthonormal with respect to $p(||\mathbf{x}||)$. After solving these radial eigenvalue problems, we obtain the following Mercer decomposition of the kernel

$$K(\mathbf{x},\mathbf{x}') = \sum_{zkm}\eta_{z,k}R_{z,k}(||\mathbf{x}||)R_{z,k}(||\mathbf{x}'||)Y_{km}(\hat{\mathbf{x}})Y_{km}(\hat{\mathbf{x}}'), \tag{95}$$

where $\eta_{z,k}$ are the eigenvalues of this decomposition, $R_{z,k}(||\mathbf{x}||)$ denotes the radial dependence and Y_{km} are hyper-spherical harmonics in D-dimensions. The eigenvalues $\eta_{z,k}$ are the same for every m for each (z,k) mode. There are at least $N(D,k) = \begin{pmatrix} k+D-1 \\ D-1 \end{pmatrix} - \begin{pmatrix} k+D-3 \\ D-1 \end{pmatrix} \sim \mathcal{O}(D^k)$ degeneracy of each kernel mode just due to the rotational symmetry (see Supplementary Note 6). In case of eigenvalue degeneracy, in order to keep $K(\mathbf{x},\mathbf{x}') \sim \mathcal{O}_D(1)$, each (z,k) term in the Mercer decomposition must be $\mathcal{O}_D(1)$. Since the sum over $N(D,k)$ orders m gives a scaling of each (z,k) term of $\mathcal{O}_D(N(D,k)) \sim \mathcal{O}_D(D^k)$, the eigenvalues must scale like $\eta_{z,k} \sim \mathcal{O}_D(1/N(D,k)) \sim \mathcal{O}_D(D^{-k})$. This observation guides us to consider another possible scaling of P via $P/N(D,k) \sim \mathcal{O}(1)$ leading to a non-trivial generalization error in the limit $P, D \to \infty$.

For the most general case, generalization error for a rotational invariant kernel is given by Supplementary Equation 53 with ρ summation replaced by a sum over (z,k,m). As noted above, eigenvalues $\eta_{z,k,m} = \eta_{z,k}$ are the same for $m = 1,...,N(D,k)$. For given k, $\eta_{z,k}$ for all z might be different with same degeneracy $N(D,k)$. In this case, we choose the scaling $\alpha = P/N(D,l) \sim \mathcal{O}_D(1)$ and take the limit $P, D \to \infty$. We find that the resulting generalization decouples over different k modes for a general target function $\bar{f}(\mathbf{x}) = \sum_{z,k,m}\bar{w}_{z,k,m}\phi_{z,k,m}(\mathbf{x})$:

$$\begin{aligned} E_g &= \frac{\kappa^2}{1-\gamma}\sum_z \frac{\bar{\eta}_{z,l}\bar{w}_{z,l}^2}{\left(\kappa+\alpha\bar{\eta}_{z,l}\right)^2} + \frac{1}{1-\gamma}\sum_{z,k>l}\bar{\eta}_{z,k}\bar{w}_{z,k}^2 + \sigma^2\frac{\gamma}{1-\gamma}, \\ \kappa &= \lambda + \sum_z \frac{\kappa\bar{\eta}_{z,l}}{\kappa+\alpha\bar{\eta}_{z,l}} + \sum_{z,k}\bar{\eta}_{z,k}, \quad \gamma = \sum_z \frac{\alpha\bar{\eta}_{z,l}^2}{(\kappa+\alpha\bar{\eta}_{z,l})^2}, \end{aligned} \tag{96}$$

where we defined the following $\mathcal{O}(1)$ quantities:

$$\bar{\eta}_{z,k} \equiv N(D,k)\eta_{z,k}, \qquad \bar{w}_{z,k}^2 \equiv \frac{1}{N(D,k)}\sum_{m=1}^{N(D,k)}\bar{w}_{z,k,m}^2. \tag{97}$$

First term corresponds to learning the mode l features while second term corresponds to the higher modes. Note that $\gamma(\alpha=0) = \gamma(\alpha=\infty) = 0$ meaning that the modes $k > l$ are not being learned in the learning stage l. Last term is the noise contribution to E_g. Furthermore, self-consistent equation for κ simplifies to a polynomial equation of degree $\#(z)+1$ instead of degree $\#(z)+\#(l)+1$, where $\#(z)$ and $\#(l)$ denote the total number of z and l modes, respectively.

We found that eigenvalues with different degeneracies $N(D,k)$ decouple as different learning stages for generic rotation invariant kernels in $D \to \infty$ limit. However, kernels with further symmetries such as translational invariance can have eigenvalues with larger degeneracies. To take this case into account, we introduce the following notation: η_K denotes the degenerate eigenvalues indexed by an

integer K potentially representing different combinations of (z,k) and $\phi_{K,\rho}$ denotes the corresponding eigenfunctions where ρ denotes collectively the degenerate indices. In this case, the degeneracy of each mode K is denoted by $N(D,K)$ which can be larger than the degeneracy of spherical harmonics. Considering the case where there is only a single eigenvalue for integer mode K with degeneracy $N(D,K)$, self-consistent equation for κ for learning stage L in Supplementary Equation 96 becomes a quadratic equation and we obtain the following solution:

$$\begin{aligned}
&\tilde{\kappa}(\alpha) \equiv \frac{\kappa}{\bar{\eta}_L} = \frac{1}{2}(1+\tilde{\lambda}_L-\alpha) + \frac{1}{2}\sqrt{(1+\tilde{\lambda}_L+\alpha)^2-4\alpha}, \\
&\tilde{\lambda}_L = \frac{\lambda + \sum_{K>L}\bar{\eta}_K}{\bar{\eta}_L}, \\
&\tilde{\kappa}(0) = 1+\tilde{\lambda}_L, \qquad \tilde{\kappa}(\infty) = \tilde{\lambda}_L, \qquad \tilde{\kappa}(\alpha) \geq 0, \ \ \forall \alpha \in \mathbb{R}^+,
\end{aligned} \tag{98}$$

where $\tilde{\kappa}$ is the scaled κ by $\bar{\eta}_K$ and $\bar{\eta}_K = N(D,K)\eta_K$. In this case, we choose the scaling $P = \alpha N(D,K)$. This formula is same as the white band-limited example except for a more complicated *effective regularization* $\tilde{\lambda}_L$. Therefore, each learning stage behaves in the same way as white band-limited case, and in the presence of noise, we may observe to see multiple descents associated to each learning episode.

Similar to the discussion for white band-limited case, $\tilde{\kappa}$ is a monotonically decreasing function of α. Effective regularization $\tilde{\lambda}_L$ controls the decay rate of $\tilde{\kappa}$ and is completely fixed by kernel eigenspectrum and explicit ridge parameter. For larger $\tilde{\lambda}_L$, the decay of $\kappa(\alpha)$ is slower and for $\tilde{\lambda}_L = 0$, decay is fastest. In fact, for the special case $\tilde{\lambda}_L = 0$ decay rate is discontinuous and the second derivative of $\tilde{\kappa}$ diverges at $\alpha = 1+\tilde{\lambda}_L = 1$.

With these definitions, γ becomes:

$$\gamma = \frac{\alpha}{(\tilde{\kappa}+\alpha)^2}. \tag{99}$$

Similar to the discussion in white band-limited example, the function γ has a maximum at $\alpha = 1+\tilde{\lambda}_L$, and as $\tilde{\lambda}_L \to 0$, its maximum goes to $\gamma \to 1$, while for large $\tilde{\lambda}_L$ its maximum falls like $1/4\tilde{\lambda}_L$. Therefore, for certain cases we expect local maxima or divergences in generalization error due to the factor of $1/(1-\gamma)$ and for larger $\tilde{\lambda}_L$ we expect the effect of peaks to decrease, acting as an effective regularization [15].

Replacing these definitions in Supplementary Equation 53, we obtain the generalization error for rotation invariant kernels as:

$$\frac{E_g^{(L)}(\alpha) - E_g^{(L)}(\infty)}{\bar{\eta}_L \bar{w}_L^2} = \frac{1}{1-\gamma}\frac{\tilde{\kappa}^2}{(\tilde{\kappa}+\alpha)^2} + \left(\frac{\sigma^2 + E_g^{(L)}(\infty)}{\bar{\eta}_L \bar{w}_L^2}\right)\frac{\gamma}{1-\gamma}, \tag{100}$$

where $E_g^{(L)}(\infty) = \sum_{K>L}\bar{\eta}_K \bar{w}_K^2$ is the asymptotic value of the generalization error and superscript (L) indicates that we are considering the scaling $P = N(D,L)\alpha$. The particular form we presented $E_g^{(L)}$ is useful to study α dependence of generalization error across different modes L since the right-hand side of the equation functionally depends only on α and $\tilde{\lambda}_L$ which is completely fixed by the full spectrum of RKHS. Asymptotically, first term is monotonically decreasing with $\frac{1}{\alpha^2}$, while the second term has a maximum at $\alpha = 1+\tilde{\lambda}_L$ with magnitude:

$$\frac{\gamma(\tilde{\lambda}_L)}{1-\gamma(\tilde{\lambda}_L)} = \frac{1}{2\sqrt{\tilde{\lambda}_L}}\frac{1}{\sqrt{\tilde{\lambda}_L}+\sqrt{1+\tilde{\lambda}_L}}, \tag{101}$$

where generalization error might display a peak with increasing training samples. Therefore, we conclude that the "double descent" behavior can only arise due to the noise in target, consistent with the observations of [16]. We also observe that the *effective noise* is given by $\tilde{\sigma}_L^2 \equiv \frac{\sigma^2 + E_g^{(L)}(\infty)}{\bar{\eta}_L \bar{w}_L^2}$ which implies that the errors from higher modes might act like noise in generalization error. Note that effective noise can be scale $N(D, L)$ dependent due to the weight factor in the denominator.

From the particular form of generalization error in Supplementary Equation 96, we observe that there is a trade-off between noiseless and the noisy term and it is not obvious for which combinations of $\tilde{\sigma}_L^2$ and $\tilde{\lambda}_L$ the generalization error has a local maximum. Similar to the discussion in white band-limited case, we obtain the a phase diagram by identifying where on the $(\tilde{\lambda}_L, \tilde{\sigma}_L^2)$ plane the first derivative of Supplementary Equation 96 vanishes defined:

$$\begin{aligned} \tilde{\sigma}_L^2 &\geq g(\tilde{\lambda}_L) \equiv 3\tilde{\lambda}_L\big(3\tilde{\lambda}_L + 2 - 2\sqrt{1+\tilde{\lambda}_L}\sqrt{9\tilde{\lambda}_L + 1}\cos\theta_L\big), \\ \theta_L &= \frac{1}{3}\left(\pi + \tan^{-1}\frac{8\sqrt{\tilde{\lambda}_L}}{9\tilde{\lambda}_L(3\tilde{\lambda}_L + 2) - 1}\right) \end{aligned} \tag{102}$$

Above this curve where non-monotonicity occurs, we further observe that the curve $\tilde{\sigma}_L^2 = 2\tilde{\lambda}_L + 1$ for $\tilde{\lambda}_L < 1$ separates two regions with a single and double local extrema.

Here, similar to the white band-limited case, we find an optimal $\tilde{\lambda}_L^* = \tilde{\sigma}_L^2$ for each learning episode L, achieving the minimum generalization error for all α.

This analysis allows us to understand the dependence of non-monotonic learning behavior on the kernel spectrum by studying $\tilde{\lambda}_L$. Let us consider the case where $\bar{\eta}_L \sim \mathcal{O}(s^{-L})$ for some $s > 1$, the case relevant for the Gaussian kernel example. Then in the ridgeless ($\lambda = 0$) limit $\tilde{\lambda}_L$ is given by:

$$\tilde{\lambda}_L = \frac{\sum_{K>L} s^{-K}}{s^{-L}} = \sum_{K=L}^{\infty} s^{-K} = \frac{1}{s-1}. \tag{103}$$

Here $\tilde{\lambda}_L$ is the same for all L. We observe that as spectrum decays faster, generalization error might feature larger peaks since the regularization $\tilde{\lambda}_L \to 0$. Therefore, faster decaying spectra are more likely to cause non-monotonic learning curves than the slower decaying ones.

Another example is $\bar{\eta}_L \sim \mathcal{O}(L^{-s})$ which is more relevant for studying neural networks. In this case, we have:

$$\begin{aligned} \tilde{\lambda}_L &= \frac{\lambda}{\bar{\eta}_L} + \frac{\sum_{K=L}^{\infty}\bar{\eta}_K}{\bar{\eta}_L} - 1 = \frac{\lambda}{\bar{\eta}_L} + \sum_{K=0}^{\infty}\left(\frac{L+K}{L}\right)^{-s} - 1 \\ &= L^s\big(\zeta(s, L) + \lambda\big) - 1, \end{aligned} \tag{104}$$

where $\zeta(s, L)$ is the Hurwitz zeta function defined as:

$$\zeta(s, L) \equiv \sum_{K=0}^{\infty}\frac{1}{(L+K)^s} = \frac{1}{\Gamma(s)}\int_0^{\infty}\frac{e^{-Lx}x^{s-1}}{1-e^{-x}}dx = \frac{l^{-s+1}}{\Gamma(s)}\int_0^{\infty}\frac{e^{-x}x^{s-1}}{1-e^{-x/L}}dx, \tag{105}$$

where in the last step, we performed the change of variables $x \to tx$. To understand the spectrum dependence of $\tilde{\lambda}_L$, we approximate $\zeta(s, L) \approx L^{-s+1}/(s-1)$ for large L. Then $\tilde{\lambda}_L$ simplifies to:

$$\tilde{\lambda}_L \approx \frac{L}{s-1} + \lambda L^s. \tag{106}$$

Similar to exponential spectrum, again the regularization falls as spectrum decays faster. Furthermore, we can see that regularization $\tilde{\lambda}_L$ increases at least linearly with L (or with power law for $\lambda \neq 0$) meaning that non-monotonicity becomes less visible for higher modes. Another note is that one can think of the quantity $\tilde{\lambda}_L$ as an "effective ridge parameter" which regularizes higher order modes causing them not to fit random noise and therefore stay smoother.

This can be thought of as an example of implicit regularization in learning machines where more complicated features (higher modes) are implicitly chosen not to be learned, since learning rates also slow down with sample complexity as $\tilde{\lambda}_L$ gets larger. This property of the power law spectrum keeps the learned function smoother. It can be also interpreted as explicitly regularizing the learning with a mode dependent ridge parameter $\lambda = L/(s-1)$.

Next, we consider concrete examples of the theory.

Gaussian kernel

As a popular example, we study Gaussian kernel which further also possesses translational symmetry. Let $p(\mathbf{x}) = \mathcal{N}(0, r^2\mathbf{I})$ be the data distribution on the input space $\mathbb{R}^D$ and $K(\mathbf{x}, \mathbf{x}') = e^{-\frac{1}{2D\omega^2}||x-x'||^2}$ be the Gaussian kernel. For this density and the kernel, the eigenfunctions and eigenvalues can be computed exactly [1]:

$$\eta_{\mathbf{k}} = \left(\frac{2a}{A}\right)^{\frac{D}{2}} \left(\frac{b}{A}\right)^{\sum_i k_i}, \quad \phi_{\mathbf{k}}(\mathbf{x}) = e^{-(c-a)\|\mathbf{x}\|^2} \prod_{i=1}^{D} H_{k_i}(\sqrt{2c}x_i), \tag{107}$$

where $a = \frac{1}{4r^2}$, $b = \frac{1}{2D\omega^2}$, $c = \sqrt{a^2 + 2ab}$ and $A = a + b + c$. The degeneracy of each mode for fixed $K = \sum_i k_i$ is given by: $\binom{K+D-1}{K} \sim \frac{D^K}{K!}$ in large D limit. We note that this system can also be thought of as a collection of D harmonic oscillators, where the eigenfunctions represent different microstates. The degeneracy in the eigenspectrum is analogous to the number of states with the same total energy: the number of distinguishable macrostates possible when K energy quanta are distributed over D oscillators. The Gaussian can be decomposed in spherical polar coordinates in terms of angular and radial functions as in Supplementary Equation 95, but this decomposition is more complicated than the decomposition in Cartesian coordinates we study here.

An informative limit to study the spectrum is one where $\omega^2 \sim \mathcal{O}_D(1)$ in the $D \to \infty$ limit. In this large D limit, the normalized spectrum converges to

$$\bar{\eta}_K = \eta_K N(D, K) \sim \left(\frac{r^2}{\omega^2 D}\right)^K \frac{D^K}{K!} = \frac{1}{K!}\left(\frac{r^2}{\omega^2}\right)^K \sim \mathcal{O}_D(1). \tag{108}$$

We can also compute the effective regularization at each learning stage

$$\begin{aligned} \tilde{\lambda}_K &= K! \left(\frac{r^2}{\omega^2}\right)^{-K} \sum_{\ell > K} \frac{1}{\ell!}\left(\frac{r^2}{\omega^2}\right)^{\ell} = \sum_{\ell=1}^{\infty} \frac{K!}{(\ell+K)!}\left(\frac{r^2}{\omega^2}\right)^{\ell} \\ &= K! \left(\frac{\omega^2}{r^2}\right)^K \left[\exp\left(\frac{r^2}{\omega^2}\right) - \sum_{\ell=0}^{K} \frac{1}{\ell!}\left(\frac{r^2}{\omega^2}\right)^{\ell}\right], \end{aligned} \tag{109}$$

which we see is a monotonically increasing function of r^2/ω^2. Thus, for fixed distribution variance r^2, a larger kernel bandwidth ω^2 leads to lower effective regularization. This is associated with larger learning curve peaks in the presence of noise. A smaller kernel bandwidth leads to larger effective regularization, mitigating the peak. The optimal ω^2 for the first learning stage can be determined

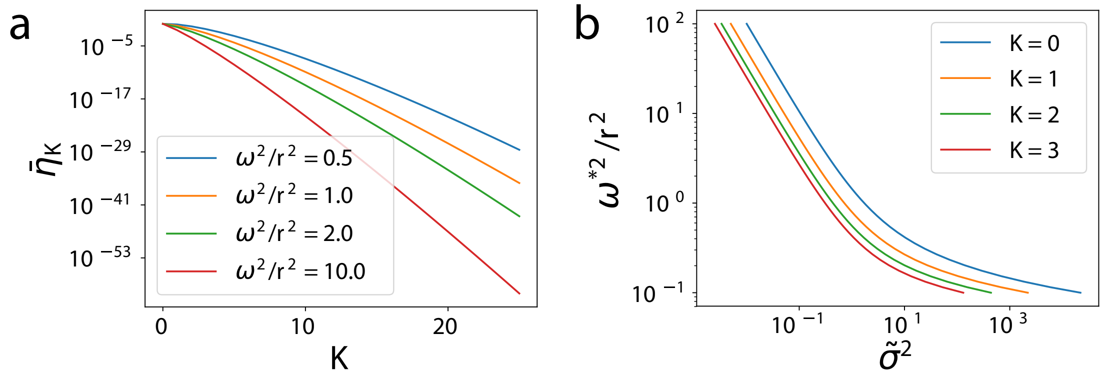

Supplementary Figure 4: Kernel Eigenspectra for the Gaussian RBF on a Gaussian measure in the $D \to \infty$ limit. **a.** Larger bandwidth spectra decay more rapidly with increasing K. **b.** The optimal bandwidth ω^* as a function of the effective noise $\tilde{\sigma}^2$. Small bandwidth kernels are preferred for late learning stages (large K) and large effective noise $\tilde{\sigma}^2$. For small $\tilde{\sigma}^2$ the optimal bandwidth satisfies $\omega^{*2} \propto \tilde{\sigma}^{-2}$ as predicted by the approximation obtained in the $r \ll \omega$ limit.

by setting $\tilde{\sigma}_K^2 = \tilde{\lambda}_K$. Under the assumption that the kernel bandwidth is large $\omega^2 \gg r^2$, we find that $\tilde{\lambda}_K \sim \frac{1}{1+K}\frac{r^2}{\omega^2}$ so that the optimal bandwidth for learning stage K and noise level $\tilde{\sigma}_K^2 \ll 1$ is

$$\omega_K^{*\,2} \sim \frac{r^2}{\tilde{\sigma}^2(K+1)}\ , \ \tilde{\sigma}^2 \to 0. \tag{110}$$

The effective eigenvalues $\overline{\eta}_K$ for each learning stage K and different bandwidths are provided in Supplementary Figure 4a. In the infinite dimension limit $D \to \infty$ the effective regularization is controlled by the bandwidth of the kernel resulting in an optimum ω^* which is plotted in Supplementary Figure 4b.

Supplementary Figure 5 displays kernel regression on a target function:

$$\bar{f}(\mathbf{x}) = \sum_{i=1}^{P'} \alpha_i K(\mathbf{x}, \bar{\mathbf{x}}_i), \quad \alpha_i \sim \mathcal{B}(1/2), \quad \bar{\mathbf{x}}_i \sim \mathcal{N}(0, \sigma^2 \mathbf{I}), \tag{111}$$

where K is the Gaussian kernel with variance ω^2 and α_i are drawn from a Bernoulli distribution. Generating P noisy labels from this function, we perform kernel regression and calculate generalization error on a randomly generated test data. We repeat this process many times to obtain training and target dataset averaged generalization error (see Supplementary Note 5 for simulation details). Kernel regression experiment fits the theory prediction almost perfectly as can be seen from Supplementary Figure 5.

Dot-Product Kernels and Neural Tangent Kernel

Here we consider the application of the generalization error Supplementary Equation 53 on dot-product kernels $K(\mathbf{x} \cdot \mathbf{x}') : \mathcal{S}^D \times \mathcal{S}^D \to \mathbb{R}$. Natural orthonormal basis on the input space $\mathcal{S}^D$ are D-dimensional hyper-spherical harmonics $\phi_\rho(\mathbf{x}) \equiv Y_{lm}(\mathbf{x})$ with $l = 0, 1, 2, 3, ...$ and $m = 1, .., N(D, l)$ where $N(D, l) = \binom{n+D-1}{n} - \binom{n+D-3}{n-2}$ is the number of degenerate modes associated to each mode l.

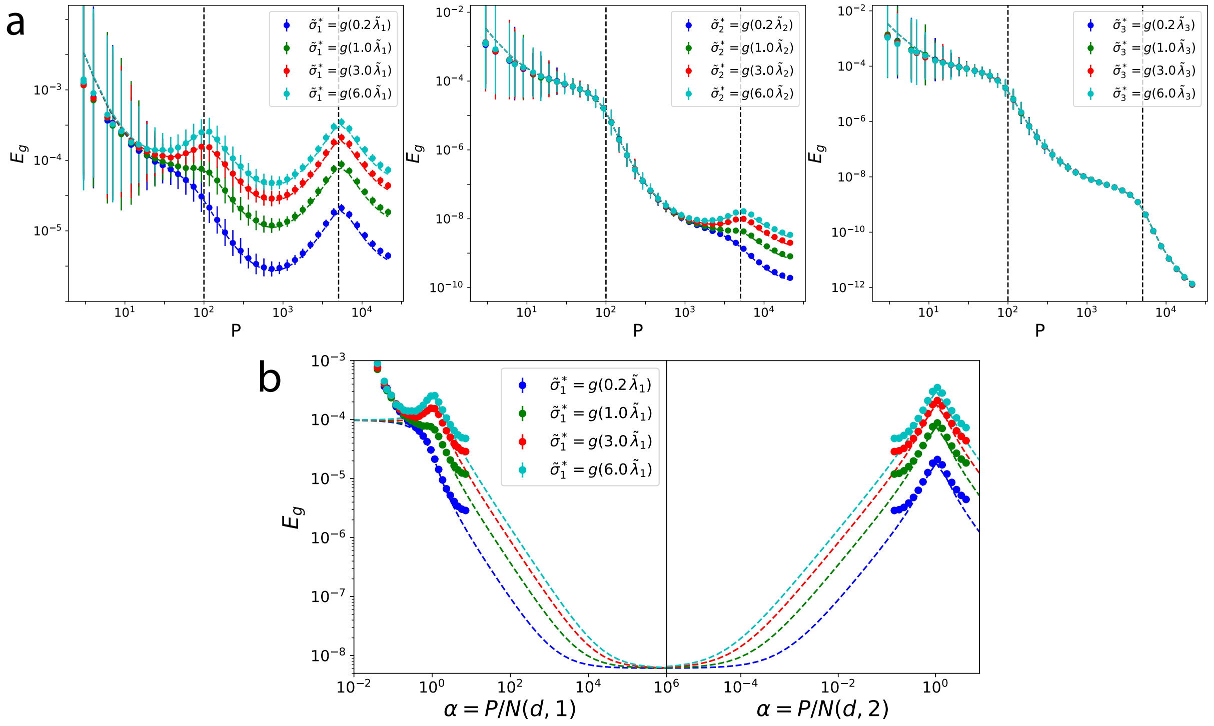

Supplementary Figure 5: Kernel regression with Gaussian RBF kernel with $\omega^2 = 900$, $\sigma = 1$ and $D = 100$ where the target was chosen as described in the Supplementary Note 5. **a.** Shows the theory curves generated by the finite P generalization error formula. Vertical dashed lines indicate different degeneracies $N(D, K)$ corresponding to learning stages $K = 1, 2$. Different panels correspond to noise levels chosen based on $\tilde{\lambda}_L$ of mode L. Error bars indicate the standard deviation over 100 trials. **b.** Same experiment (left panel in **a**) for varying $\tilde{\sigma}_1$ compared to the $P, D \to \infty$ version of the generalization error formula. Around $P \sim N(D, L)$ for each learning stage, E_g obtained above still predicts very well except in the middle regions, finite P, D effects dominate. Error bars are removed for visualization.

We consider kernel ridgeless regression ($\lambda = 0$) with a kernel with power law spectrum $\bar{\eta}_k = k^{-s}$. In this case, effective regularization $\tilde{\lambda}_l$ increases with mode l since $\tilde{\lambda}_l \approx l/(s-1)$. With a similar procedure applied in Gaussian RBF example, we set target weights $\bar{w}_k^2 = \eta_k = \bar{\eta}_k / N(D,k)$. A kernel regression experiment and prediction are shown in Supplementary Figure 6a. Note that we use the finite P version of the generalization error to produce this plot meaning that the theory is still perfectly predictive without taking the infinite P limit. In each panel, the label noise variance chosen according to $g(\tilde{\lambda}_k)$ corresponding to the learning stage $k = 1, 2, 3$. When $\tilde{\sigma}_k^2 < g(\tilde{\lambda}_k)$, generalization error falls monotonically at the learning stage k and when $\sigma^2 > g(\tilde{\lambda}_k)$ we observe double-descent. Supplementary Figure 6b, on the other hand, demonstrates how infinite P and D limit of E_g, Eq. (9) in main text, predicts the regression experiments at each learning stage (corresponding to different panels) when P is finite. We observe that a later learning stage starts before the previous learning stage saturates to its asymptotic value.

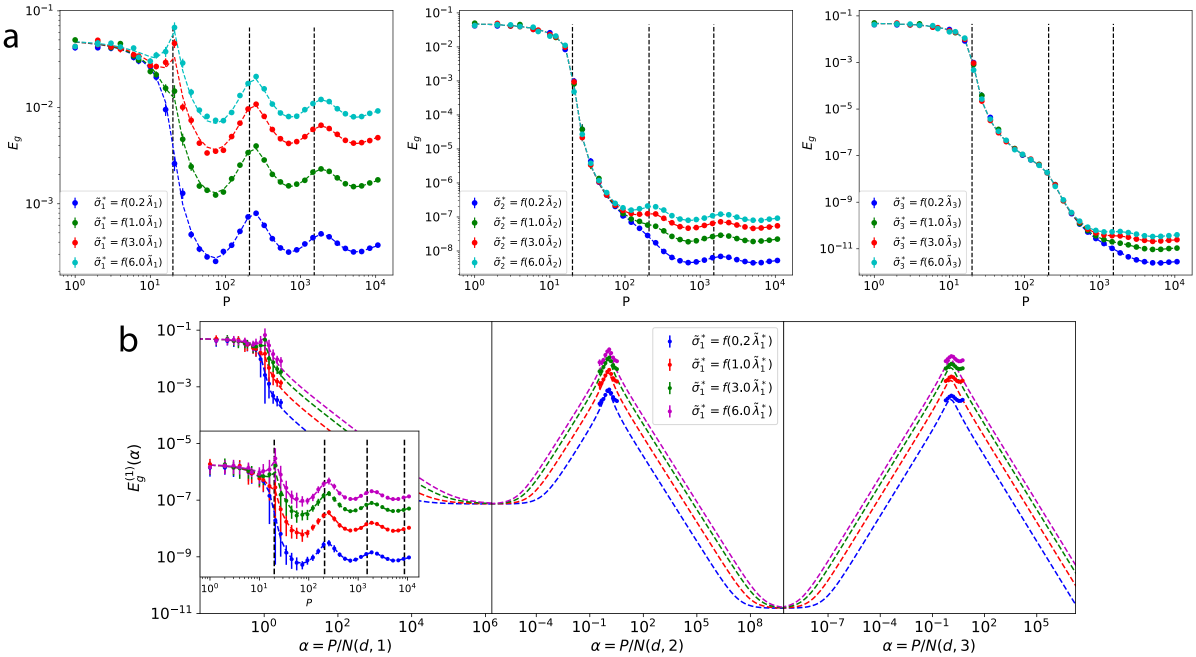

Supplementary Figure 6: Kernel ridgeless regression with power law kernel $\bar{\eta}_k = k^{-8}$ and $D = 20$ where the target was chosen as described in the Supplementary Note 5. **a.** Shows the theory curves generated by the generalization error formula given in Eq. (4) in main text evaluated at finite P. Vertical dashed lines indicate different degeneracies $N(D,l)$ corresponding to learning stage l. Different panels correspond to noise levels chosen based on $\tilde{\lambda}_l$ of mode l. Error bars indicate the standard deviation over 30 trials. **b.** Same experiment for varying $\tilde{\sigma}_1$ compared to the $P, D \to \infty$ version of the generalization error formula, Eq. (9) in main text. E_g still predicts experiments very well around $P \sim N(D,l)$ for each learning stage. Finite P, D effects dominate beyond. Error bars are removed for visualization.

The relevance of power law spectra and dot-product kernels to deep neural networks comes from the correspondence of infinitely wide neural networks and ridgeless kernel regression [17]. Consider a neural network with L hidden layers where $n_\ell = n$ units in each of these layers for $\ell = 1, \ldots, L$ and $n_0 = D$ being the input dimension. We initialize the weights in each layer randomly $W_{ij}^{(\ell)} \sim \mathcal{N}(0, \sigma_W^2)$. With this parameterization and the concatenation of all network parameters in

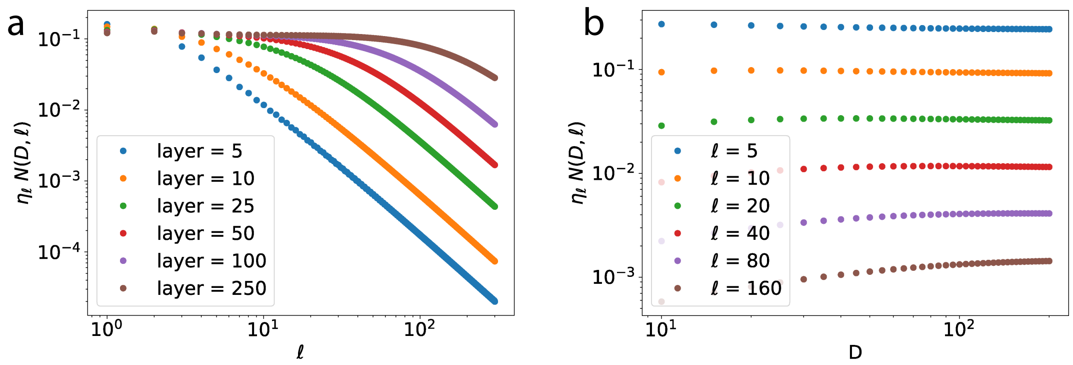

Supplementary Figure 7: Spectrum dependence of NTK to number of layers and input dimension. **a.** Empirically, spectrum $\bar{\eta}_l = \eta_l N(D,l)$ becomes white as more layers added. **b.** Furthermore, we confirm that the spectrum $\bar{\eta}_l$ is independent of input dimension for large D.

$\boldsymbol{\theta} = \mathrm{Vec}\{\mathbf{W}^{(\ell)}\}_{\ell=1}^{L+1}$, the network function is:

$$f(\mathbf{x};\boldsymbol{\theta}) = n^{-1/2}\mathbf{W}^{(L+1)}\sigma\left(n^{-1/2}\mathbf{W}^{(L)}\sigma\left(\dots n^{-1/2}\mathbf{W}^{(2)}\sigma\left(n_0^{-1/2}\mathbf{W}^{(1)}\mathbf{x}\right)\right)\right), \tag{112}$$

where σ is a non-linearity. We will only consider the Rectified Linear Unit (ReLU). Training the network parameters $\boldsymbol{\theta}$ with gradient flow on a squared loss to zero training error is equivalent to the function obtained from ridgeless kernel regression with the Neural Tangent Kernel (NTK) [17, 18, 19]. This kernel can be obtained heuristically by linearizing the neural network function $f(\mathbf{x},\boldsymbol{\theta})$ around its initial set of parameters $\boldsymbol{\theta}_0$, $f(\mathbf{x},\boldsymbol{\theta}) \approx f(\mathbf{x},\boldsymbol{\theta}_0) + \nabla_{\boldsymbol{\theta}} f(\mathbf{x},\boldsymbol{\theta}_0)\cdot(\boldsymbol{\theta}-\boldsymbol{\theta}_0)$. Optimizing a mean squared regression error over $\boldsymbol{\theta}$ is equivalent to solving a linear regression problem for $\boldsymbol{\theta}$ where the feature Gram matrix is formed from initial parameter gradients: $\mathbf{K}_{\mathrm{NTK},ij} = \nabla_{\boldsymbol{\theta}} f(\mathbf{x}_i,\boldsymbol{\theta}_0)\cdot\nabla_{\boldsymbol{\theta}} f(\mathbf{x}_j,\boldsymbol{\theta}_0)$. In the infinite-width limit, this quantity converges to its average over all possible initializations $\boldsymbol{\theta}_0$, giving rise to the deterministic NTK [17]. As an example, the exact form of NTK for ReLU non-linearity and zero bias is given by:

$$\begin{aligned}
K^{(0)}_{\mathrm{NTK}}(\mathbf{x},\mathbf{x}') &= \cos^{-1}(\mathbf{x}\cdot\mathbf{x}')\\
K^{(1)}_{\mathrm{NTK}}(\mathbf{x},\mathbf{x}') &= \cos\left[f\left(\cos^{-1}(\mathbf{x}\cdot\mathbf{x}')\right)\right] + K^{(0)}_{\mathrm{NTK}}(\mathbf{x},\mathbf{x}')\left(1-\frac{\cos^{-1}(\mathbf{x}\cdot\mathbf{x}')}{\pi}\right)\\
K^{(2)}_{\mathrm{NTK}}(\mathbf{x},\mathbf{x}') &= \cos\left[f\left(f\left(\cos^{-1}(\mathbf{x}\cdot\mathbf{x}')\right)\right)\right] + K^{(1)}_{\mathrm{NTK}}(\mathbf{x},\mathbf{x}')\left(1-\frac{f\left(\cos^{-1}(\mathbf{x}\cdot\mathbf{x}')\right)}{\pi}\right)\\
&\dots\\
K^{(L)}_{\mathrm{NTK}}(\mathbf{x},\mathbf{x}') &= \cos\Big[\underbrace{f(f(f(\dots f(\cos^{-1}(\mathbf{x}\cdot\mathbf{x}')))))}_{L\text{ times}}\Big] + K^{(L-1)}_{\mathrm{NTK}}(\mathbf{x},\mathbf{x}')\left(1-\frac{\overbrace{f(f(f(\dots f(\cos^{-1}(\mathbf{x}\cdot\mathbf{x}')))))}^{L-1\text{ times}}}{\pi}\right),
\end{aligned} \tag{113}$$

where $f(\theta) = \cos^{-1}\left[\frac{1}{\pi}\left(\sin(\theta) + \left(\pi-\theta\right)\cos(\theta)\right)\right]$. By projecting this function onto the Gegenbauer polynomials, we can obtain the spectrum of NTK for any layer [12]. We empirically observe that the eigenvalues obey power-law for large modes as seen from Supplementary Figure 7.

Having obtained the kernel and its spectrum, we perform kernel regression with the exact infinite-width limit NTK and train the corresponding finite width neural network. In Supplementary

Figure 8a, we demonstrate the results for fitting a pure mode target function $\bar{f}(\mathbf{x}) = a_k Q_k^{(D-1)}(\boldsymbol{\beta} \cdot \mathbf{x})$ which has vanishing weights except for a single mode k. $\boldsymbol{\beta}$ is randomly generated. We find that our theory describes NTK regression perfectly while neural network experiments show deviation from the theory at large P, possibly due to finite size effects. Indeed, increasing the width leads to a better match between experimental neural network risk and our theory, as shown in Supplementary Figure 8b.

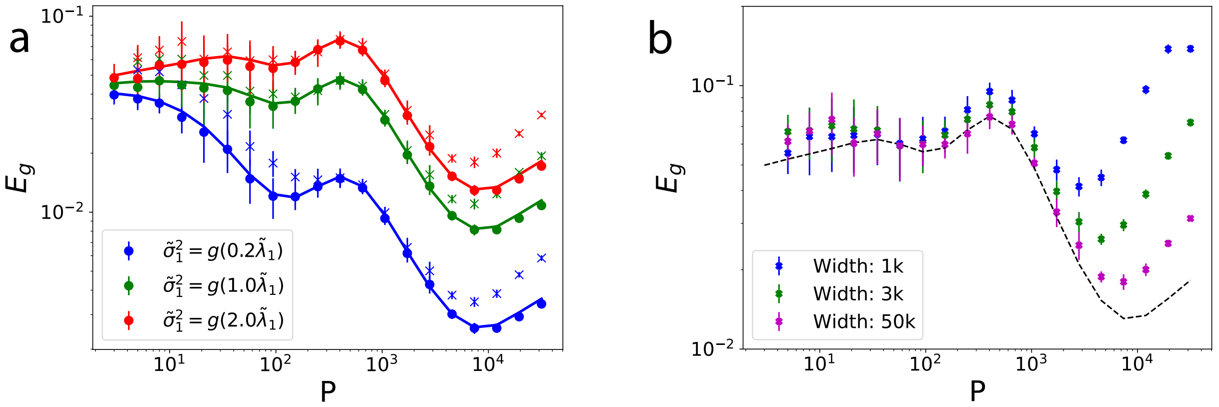

Supplementary Figure 8: **a.** 2-layer NTK regression and corresponding neural network training with 50000 hidden units for $D = 25$ with varying noise levels. Target function is the same as Fig. 6 in main text and is explained in detail in Supplementary Note 5. Solid lines are the theory predicted learning curves, dots represent NTK regression and $\times$ represents E_g after neural network training. **b.** Generalization error for 2-layer NN with varying hidden units. We observe that increasing the width brings the learning curve closer to the NTK regression theory (dashed lines). Error bars represent standard deviation for kernel regression and neural network experiment over 15 and 5 trials, respectively.

We further observe a discrepancy between NTK regression and neural network training at low-P where the generalization error is systematically biased towards higher values than its kernel regression counterpart. While this effect requires a further study of the correspondence between finite width neural networks and NTK regression, and remains to be fully understood, we empirically observe that the method of neural network ensembling [20, 16, 9] removes this discrepancy (Supplementary Figure 9b). Neural network ensembling is known to reduce the variance due to random parameter initializations [16] and hence also prevents the high-P mismatch we observe in Fig. 6 in main text and Supplementary Figure 9a.

Approximate Scaling of Learning Curves

A coarse approximation to our derived learning curves can be deduced provided that the kernel eigenspectrum obeys a certain property. The theoretical mode errors equations reveal that mode errors decay at different rates which are set by the kernel eigenvalues $\{\eta_\rho\}$. We exploit this fact to identify the number of eigenmodes that have been estimated once P samples are taken. This is equivalent to identifying the mode $\rho^*(P)$ which satisfies

$$\kappa(P) \approx P\eta_{\rho^*(P)} \tag{114}$$

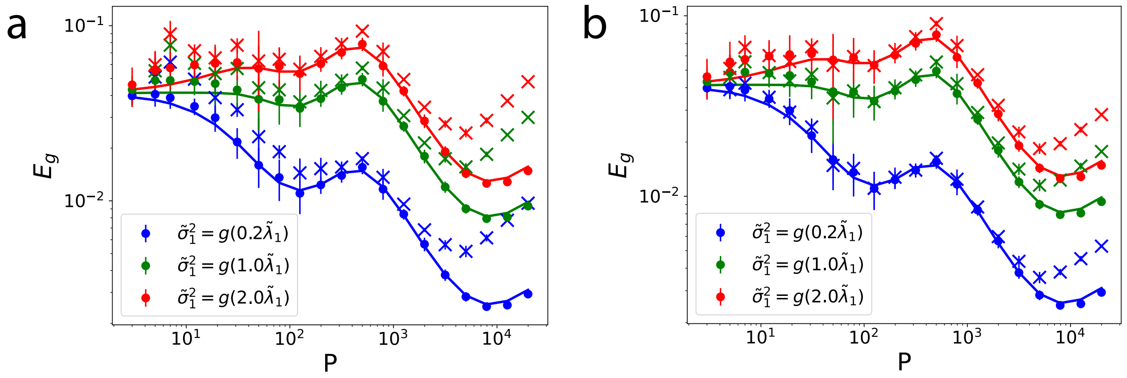

Supplementary Figure 9: **a.** 2-layer NTK regression and corresponding neural network training with 4000 hidden units for $D = 25$ with varying noise levels. Target function is the same as Fig. 6 in main text and is explained in detail in Supplementary Note 5. Solid lines are the theory predicted learning curves, dots represent NTK regression and $\times$ represents E_g after neural network training. Notice the mismatch between regression and neural network experiment for low P regime. **b.** Same experiment with 10 times ensembling. We observe that neural network ensembling reduces the mismatch for both low and high P regimes. Error bars represent standard deviation for kernel regression and neural network experiment over 15 and 5 trials, respectively.

If $\kappa \gg P\eta_\rho$ then mode ρ is not yet being learned while $P\eta_\rho \gg \kappa$ implies that the mode ρ has already been estimated accurately. The self-consistency condition for κ in the $\lambda \to 0$ limit is

$$1 = \sum_\rho \frac{\eta_\rho}{\eta_\rho P + \kappa} \approx \frac{1}{P} \sum_{\rho<\rho^*} \frac{\eta_\rho}{\eta_\rho} + \frac{1}{\kappa} \sum_{\rho>\rho^*} \eta_\rho = \frac{\rho^*(P)}{P} + \frac{1}{\kappa} \sum_{\rho>\rho^*} \eta_\rho. \tag{115}$$

Using the assumption that $\kappa(P) \approx P\eta_{\rho^*(P)}$, we find

$$P = \rho^* + \frac{1}{\eta_{\rho^*}} \sum_{\rho>\rho^*} \eta_\rho. \tag{116}$$

The solution $\rho^* \sim P$ is self-consistent provided that the effective regularization grows sublinearly

$$\tilde{\lambda}_\rho = \frac{1}{\eta_\rho} \sum_{\rho'>\rho} \eta_{\rho'} \sim \mathcal{O}(\rho). \tag{117}$$

This condition is not guaranteed to be satisfied, but if it is, then the scaling $\rho^*(P) \sim P$ is valid and we can approximate the remaining error with the power in all modes $\rho > \rho^* \approx P$

$$E_g(P) \sim \sum_{\rho>p} \eta_\rho \overline{w}_\rho^2, \tag{118}$$

which indicates that, at P, samples the expected generalization error can be approximated by a tail sum of the power in the target function. Examples of spectral decay rates that satisfy the sufficient condition above include power law $\eta_\rho \sim \rho^{-b}$ and exponential decays $\eta_\rho \sim u^\rho$, where b and u are constants. In Supplementary Figure 10, we compare this coarse grained theory to the MNIST experiments performed and reported in Fig. 1e in main text.

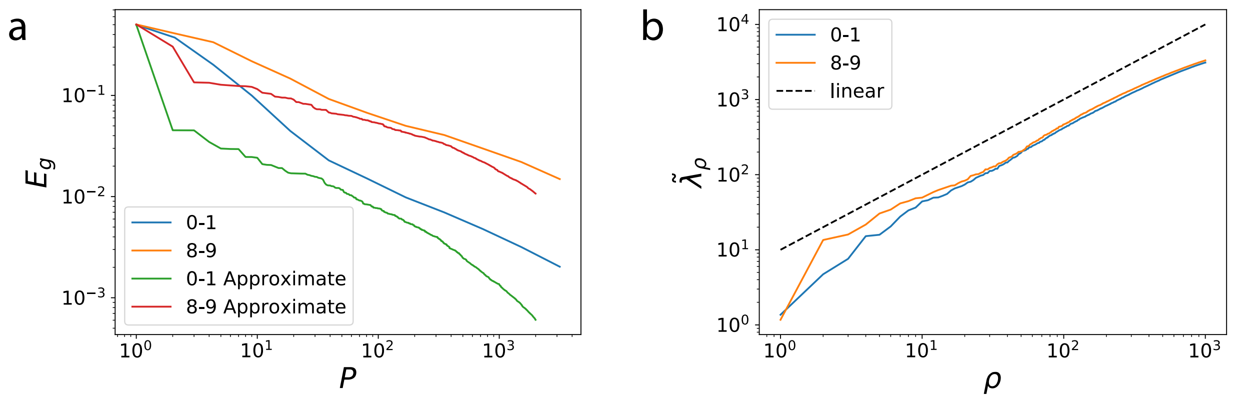

Supplementary Figure 10: **a.** Approximate Learning Curves for the Easy-Hard tasks in Fig. 1 in main text using tail sums. **b.** The effective regularization is only weakly sublinear in ρ.

Supplementary Note 5 - Details of Numerical Experiments

Calculating Kernel Spectrum for Spherical Data

For a chosen kernel, one can find the eigendecomposition onto spherical harmonics Y_{kl} and Gegenbauer polynomials $Q_k^{(\alpha)}$ [21] using the following formula:

$$K(\mathbf{x},\mathbf{y}) = \sum_{k=0}^{\infty} \eta_k \sum_{m=1}^{N(D,k)} Y_{k,m}(\mathbf{x}) Y_{k,m}(\mathbf{y}) = \sum_k \eta_k \frac{k+\alpha}{\alpha} Q_k^{(\alpha)}(\mathbf{x}\cdot\mathbf{y}). \tag{119}$$

Here $\alpha = \frac{D-2}{2}$. Projecting the kernel on the Gegenbauer basis, we obtain the eigenvalues:

$$\begin{aligned} \int_{-1}^{1} dt\,(1-t^2)^{\alpha-\frac{1}{2}} K(t) Q_k^{(\alpha)}(t) &= \sum_l \eta_l \frac{l+\alpha}{\alpha} \int_{-1}^{1} dt\,(1-t^2)^{\alpha-\frac{1}{2}} Q_l^{(\alpha)}(t) Q_k^{(\alpha)}(t) \\ &= \eta_k N(D,k) \frac{\omega_D}{\omega_{D-1}} \frac{\alpha}{k+\alpha}, \end{aligned} \tag{120}$$

where $\omega_D = \frac{2\pi^{D/2}}{\Gamma(D/2)}$ is the surface area of a unit D-sphere and $N(D,k)$ is the degeneracy (see Supplementary Note 6). Hence, the eigenvalues of the kernel are found by the integral:

$$\eta_k N(D,k) = \frac{\omega_{D-1}}{\omega_D} \frac{k+\alpha}{\alpha} \int_{-1}^{1} dt\,(1-t^2)^{\alpha-\frac{1}{2}} K(t) Q_k^{(\alpha)}(t) \tag{121}$$

We compute the eigenvalues η_l of a kernel $K(t)$ by numerically performing the integral in Supplementary Equation 121 with Gauss-Gegenbauer quadrature rule [22] for the measure $(1-t^2)^{\alpha-1/2}$. This formula is used to find the eigenspectrum of NTK in Supplementary Figure 7 and calculate the NTK learning curves in Fig. 6 in main text and in Supplementary Figure 8.

Synthetic Data Experiments on Unit Sphere

We generally consider a scalar target functions of the form:

$$\bar{f}(\mathbf{x}) = \sum_k a_k Q_k^{(\alpha)}(\boldsymbol{\omega}_k \cdot \mathbf{x}), \quad \boldsymbol{\omega}_k, \mathbf{x} \in \mathbb{S}^{D-1}, \tag{122}$$

where $\boldsymbol{\omega}_k$ is a random projection vector on $\mathbb{S}^{D-1}$ and a_k are coefficients. Using the identity $Q_k^{(\alpha)}(\boldsymbol{\omega}\cdot\mathbf{x}) = \frac{\alpha}{k+\alpha}\sum_{m=1}^{N(D,k)} Y_{km}(\boldsymbol{\omega})Y_{km}(\mathbf{x})$ [21], we can expand this target function in terms of the features $\psi_{km}(\mathbf{x}) = \sqrt{\eta_k}Y_{km}(\mathbf{x})$:

$$\begin{aligned}\bar{f}(\mathbf{x}) &= \sum_k a_k Q_k^{(\alpha)}(\boldsymbol{\omega}_k\cdot\mathbf{x}) = \sum_k\sum_{m=1}^{N(D,k)} a_k\frac{\alpha}{k+\alpha}Y_{km}(\boldsymbol{\omega}_k)Y_{km}(\mathbf{x}), \quad \boldsymbol{\omega},\mathbf{x}\in\mathbb{S}^{D-1}\\ &\equiv \sum_k\sum_{m=1}^{N(D,k)} \bar{w}_{km}\psi_{km}(\mathbf{x}), \quad \bar{w}_{km} = \frac{a_k}{\sqrt{\eta_k}}\frac{\alpha}{k+\alpha}Y_{km}(\boldsymbol{\omega}_k)\end{aligned} \tag{123}$$

Therefore, we obtain:

$$\bar{w}_k^2 \equiv \frac{1}{N(D,k)}\sum_m \bar{w}_{km}^2 = \frac{a_k^2}{\eta_k}\frac{\alpha^2}{(k+\alpha)^2} \tag{124}$$

where we used $Q_k^{(\alpha)}(1) = \frac{\alpha}{k+\alpha}N(D,k)$ [21]. Having obtained the weights, we can find the generalization error using Supplementary Equation 53:

$$E_g = \frac{\kappa^2}{1-\gamma}\sum_\rho \frac{\eta_\rho\bar{w}_\rho^2}{\left(\kappa+P\eta_\rho\right)^2} + \sigma^2\frac{\gamma}{1-\gamma} = \boxed{\frac{\kappa^2}{1-\gamma}\sum_k N(D,k)\frac{\eta_k\bar{w}_k^2}{\left(\kappa+P\eta_k\right)^2} + \sigma^2\frac{\gamma}{1-\gamma}}, \tag{125}$$

where $\kappa = \lambda + \kappa\sum_k N(D,k)\frac{\eta_k}{\kappa+P\eta_k}$ to be solved self-consistently and $\gamma = \sum_k N(D,k)\frac{P\eta_k^2}{(\kappa+P\eta_k)^2}$. Hence this formula allows us to create a wide range of target functions and calculate the theoretical generalization error for a regression task on it. In our experiments, we consider two types of target functions as detailed below.

Pure Target Functions

We consider target functions of the form $\bar{f}(\mathbf{x}) = a_k Q_k^{(\alpha)}(\boldsymbol{\omega}\cdot\mathbf{x})$ where k is an integer. These functions are expressed with a single mode and simpler to evaluate numerically. Throughout our experiments, we picked $a_k = \eta_k\frac{k+\alpha}{\alpha}$ so that $\bar{w}_k^2 = \eta_k$. Hence the generalization error is simply:

$$E_g = \frac{\kappa^2}{1-\gamma}N(D,k)\frac{\eta_k^2}{\left(\kappa+P\eta_k\right)^2} + \sigma^2\frac{\gamma}{1-\gamma}. \tag{126}$$

For synthetic neural network experiments in Fig. 6 in main text and Supplementary Figure 8, we use this type of target functions with $k=1$ (hence linear target function). The code referenced in Methods section can be used for reproducing the results in this paper as well as for experimenting with different target functions including mixed target functions.

Target Function with Non-zero Weights and Synthetic Kernel Regression Experiments

Generating a target function with all weights being non-zero with the method described above may be computationally expensive because sampling from many high dimensional spherical harmonics is not efficient. Instead, we devise the following method:

- We generate the target function using the representer's theorem. We choose P' target examples $\{\bar{\mathbf{x}}^\mu\}$ (different than the training set) on the sphere and observe that:

$$\bar{f}(\mathbf{x}) = \sum_{\mu=1}^{P'} \bar{\alpha}_\mu K(\mathbf{x}, \bar{\mathbf{x}}^\mu) = \sum_{\rho=1}^{N} \left(\sum_{\mu=1}^{P'} \bar{\alpha}_\mu \sqrt{\eta_\rho} \phi_\rho(\bar{\mathbf{x}}^\mu) \right) \sqrt{\eta_\rho} \phi_\rho(\mathbf{x}) \equiv \sum_{\rho=1}^{N} \bar{w}_\rho \psi_\rho(\mathbf{x}) \tag{127}$$

We note that $\bar{w}_\rho$ are random variables. To calculate their statistics, suppose we draw $\bar{\alpha}_\mu$ for each example i.i.d. from a distribution with mean 0 and variance $1/P'$. Then averaging over many $\{\mathbf{x}^\mu\}$ with large P', we get the mean and variance of $\bar{w}_\rho$ to be:

$$\langle \bar{w}_\rho \rangle = 0, \quad \langle \bar{w}_\rho \bar{w}_\gamma \rangle = \eta_\rho \delta_{\rho\gamma}. \tag{128}$$

For large P', $|\bar{w}_\rho|^2$ concentrates around η_ρ, which we use in our theoretical calculations.

We also allow sample corruption by a Gaussian noise:

$$y^\mu = \sum_{\mu=1}^{P} \bar{\alpha}^\mu K(\mathbf{x}, \bar{\mathbf{x}}^\mu) + \epsilon^\mu, \tag{129}$$

where noise for each sample has variance $\langle \epsilon^\mu \epsilon^\nu \rangle = \sigma^2 \delta^{\mu\nu}$.

- To solve the kernel regression problem, we again use the representer's theorem. Given P training samples, $\{\mathbf{x}^\mu\}$, the solution is of the form:

$$f(\mathbf{x}) = \sum_{\mu=1}^{P} \alpha_\mu K(\mathbf{x}, \mathbf{x}^\mu). \tag{130}$$

Plugging this into the kernel regression problem, with samples $y^\mu = \bar{f}(\mathbf{x}^\mu) + \epsilon^\mu$ generated by the target, we obtain the coefficients:

$$\min_{\boldsymbol{\alpha}} \left(\frac{1}{2} (\mathbf{y} - \mathbf{K}\boldsymbol{\alpha})^\top (\mathbf{y} - \mathbf{K}\boldsymbol{\alpha}) + \frac{\lambda}{2} \boldsymbol{\alpha}^\top \mathbf{K} \boldsymbol{\alpha} \right), \quad \Longrightarrow \quad \boldsymbol{\alpha} = (\mathbf{K} + \lambda \mathbf{I})^{-1} \mathbf{y}. \tag{131}$$

- Once we get these coefficients $\boldsymbol{\alpha}$, we can express the total generalization error as a sum of mode wise errors

$$\begin{aligned} E_g &= \left\langle (f(x) - \bar{f}(x))^2 \right\rangle \\ &= \sum_{\rho\gamma} \eta_\rho \eta_\gamma \Big[\sum_{j=1}^{P} \alpha_j \phi_\rho(x_j) - \sum_{i=1}^{P'} \overline{\alpha}_i \phi_\rho(\overline{x}_i) \Big] \Big[\sum_{j=1}^{P} \alpha_j \phi_\gamma(x_j) - \sum_{i=1}^{P'} \overline{\alpha}_i \phi_\gamma(\overline{x}_i) \Big] \Big\langle \phi_\rho(x) \phi_\gamma(x) \Big\rangle \\ &= \sum_{\rho} \eta_\rho^2 \Big[\sum_{j,j'} \alpha_j \alpha_{j'} \phi_\rho(x_j) \phi_\rho(x_j) - 2 \sum_{i,j} \alpha_j \overline{\alpha}_i \phi_\rho(x_j) \phi_\rho(\overline{x}_i) + \sum_{i,i'} \overline{\alpha}_i \overline{\alpha}_{i'} \phi(\overline{x}_i) \phi(\overline{x}_{i'}) \Big]. \end{aligned}$$

If we recognize that ρ indexes both (k, m) for the spherical harmonics, we can simplify the mode error to a simple matrix expression

$$\epsilon_k = \eta_k^2 \frac{k + \alpha}{\alpha} \Big[\boldsymbol{\alpha}^\top Q_k^{(\alpha)} (\mathbf{X}^T \mathbf{X}) \boldsymbol{\alpha} - 2 \boldsymbol{\alpha}^\top Q_k^{(\alpha)} (\mathbf{X}^T \overline{\mathbf{X}}) \overline{\boldsymbol{\alpha}} + \overline{\boldsymbol{\alpha}}^\top Q_k^{(\alpha)} (\overline{\mathbf{X}}^T \overline{\mathbf{X}}) \overline{\boldsymbol{\alpha}} \Big]. \tag{132}$$

We use this expression to compute experimental mode errors.

The theoretical generalization error can be obtained simply replacing $\bar{w}_\rho^2$ with the corresponding eigenvalue η_ρ Supplementary Equation 128 in our expression Supplementary Equation 125. Gaussian RBF regression experiments are performed with the same method.

Details of Neural Network Experiments

To perform neural network experiments, we use Neural Tangents package [23]. The label generating target functions are picked using the method for pure target functions (Supplementary Note 5). After we pick P labelled training data, we generate a two-layer neural network using NTK initialization [17] with weight variance $\sigma_W^2 = 1$ and bias variance $\sigma_b^2 = 0$ with ReLU activation. We pick hidden layer widths varying between $4,000 - 50,000$. The network is trained with ADAM optimizer with learning rate 0.008. We perform 5 times averaging, and we sample different data points at each trial.

Corresponding NTK ridgeless regression experiments performed with zero regularization and the theory is calculated using the numerically calculated NTK spectrum and picking the weights to be equal to the spectrum.

White Band-limited Kernel Experiments

The simplest kernel to experimentally probe the white bandlimited setting is the linear kernel $K(\mathbf{x}, \mathbf{x}') = \mathbf{x} \cdot \mathbf{x}'$ where $\mathbf{x} \sim \mathcal{N}(0, \mathbf{I})$. In this setting, the Mercer eigenfunctions are exactly the individual input vector elements $\phi_\rho(\mathbf{x}) = x_\rho$, $\rho = 1, ..., N$:

$$K(\mathbf{x}, \mathbf{x}') = \sum_{\rho=1}^{N} x_\rho x'_\rho \ , \quad \int x_\rho K(\mathbf{x}, \mathbf{x}') p(\mathbf{x}) d\mathbf{x} = x'_\rho \tag{133}$$

We used this kernel and a linear target function $f_t(\mathbf{x}) = \boldsymbol{\beta} \cdot \mathbf{x}$ where $\boldsymbol{\beta} \sim \mathcal{N}(0, \mathbf{I})$ in the Fig. 3 experiments in main text. The number of features hence the input dimension is $N = 500$.

The one dimensional plot of the averaged estimator for uniform data on S^1 in Fig. 4 in main text used the following kernel

$$K(x, x') = 2 \sum_{k=1}^{N} \cos\big(k(x - x')\big) = 2 \sum_{k=1}^{N} \Big[\cos(kx) \cos\big(kx'\big) + \sin(kx) \sin\big(kx'\big)\Big] \tag{134}$$

which is an RKHS with dimension $2N$ and the corresponding features are $\psi_k^{cos}(x) = \sqrt{2} \cos(kx)$ and $\psi_k^{sin}(x) = \sqrt{2} \cos(kx)$. In this experiment, the target function was chosen to be the (centered) vonMises function

$$\bar{f}(x) = e^{4(\cos(x)-1)} - I_0(4) \tag{135}$$

where I_0 is the Bessel function of order 0. The RKHS weights $\bar{a}_k$ were calculated by projecting the function on the features $\psi_k^{cos}(x)$ and $\psi_k^{sin}(x)$. To ensure the target is in the RKHS, we develop a bandlimited version

$$\bar{f}(x) = \sum_{k=1}^{N} \bar{a}_k \cos(kx). \tag{136}$$

Training data is generated using this target function by randomly choosing $x \in [-\pi, \pi]$ and the perform the kernel regression to obtain the estimator's RKHS weights. Finally, we evaluate the estimator on the interval $[-\pi, \pi]$ to compare the learned function to the target.

Supplementary Note 6 - Notes on Spherical Harmonics

Here we collect some useful results on spherical harmonics. Details can be found in [21]. We are interested in finding a basis for the functions space on $\mathbb{S}^{D-1} \subset \mathbb{R}^D$. Let $\mathcal{P}_k^D$ to be the space of

homogeneous polynomials of degree k. Then its dimension is:

$$\dim \mathcal{P}_k^D = \binom{k+D-1}{k} \tag{137}$$

Spherical harmonics are homogeneous $Y_{km}(t\mathbf{x}) = t^k Y_{km}(\mathbf{x})$, harmonic $\nabla^2 Y_{km}(\mathbf{x}) = 0$ polynomials, restricted to $\mathbb{S}^{D-1}$. They are orthonormal with respect to the uniform measure on the sphere

$$\int_{\mathbb{S}^{D-1}} Y_{km}(\mathbf{x}) Y_{k'm'}(\mathbf{x}) d\mathbf{x} = \delta_{k,k'}\delta_{m,m'} \tag{138}$$

The number of degree k spherical harmonics in dimension D is

$$N(D,k) = \binom{k+D-1}{k} - \binom{k+D-3}{k-2} = \frac{2k+D-2}{k}\binom{k+D-3}{k-1} \tag{139}$$

For large dimension $D \to \infty$ this number of degree k harmonics grows like

$$N(D,k) \sim \frac{D^k}{k!}, D \ \to \ \infty \tag{140}$$

The Gegenbauer polynomial of degree k, $Q_k^{(\alpha)}$, can be related to all of the degree k spherical harmonics

$$Q_k^{(\alpha)}(\mathbf{x}\cdot\mathbf{y}) = \frac{\alpha}{k+\alpha}\sum_{m=1}^{N(D,k)} Y_{km}(\mathbf{x})Y_{km}(\mathbf{y}), \quad \mathbf{x},\mathbf{y} \in \mathbb{R}^D, \tag{141}$$

where $\alpha = \frac{D-2}{2}$. We often refer to $Q_k^{(\alpha)}(\mathbf{x}\cdot\mathbf{y})$ as $Q_k^{(D-1)}(\mathbf{x}\cdot\mathbf{y})$ in the main text to emphasize that it is a polynomial defined on $\mathcal{S}^{D-1}$.

Supplementary Discussion

Relation to Kernel Alignment Risk Estimator (KARE)

A closely related study to our theory on the generalization of kernel methods utilizes random matrix theory to arrive at a related, but different, generalization prediction [4]. Their Kernel Alignment Risk Estimator (KARE) was shown to accurately predict generalization on the Higgs and MNIST datasets. The *signal capture threshold* (SCT) which arises in KARE theory is equivalent to the quantity we denote as κ, which can be interpreted as the resolvent of a generalized Wishart matrix (SI.3.1). The mode independent prefactor in our theory $\frac{1}{1-\gamma}$, which plays an important role in the KARE theory, can be obtained from differentiation κ with respect to the ridge $\partial_\lambda \kappa$. Despite these similarities, our theory is not equivalent to KARE, which can be easily seen in the $\lambda \to 0$ limit, in which KARE scales with P in a manner that does not depend on the kernel or task spectra whereas our learning curves still depend on η_ρ and $\overline{w}_\rho^2$ even in the ridgeless limit. Our theory works well even in the $\lambda = 0$ simulations, for example in Fig. 3a and Fig. 4a in main text.

KARE may appear easier to implement than the theory we present here, since its only operations involve taking inverses, traces, and quadratic forms of kernel Gram matrices, whereas our theory involves a full eigendecomposition. In terms of time complexity, however, both methods scale as $O(M^3)$ for a sampled dataset of size M.

Relation to Kernel Alignment

In this paper, we introduced the idea of task-model alignment. A similar notion of kernel compatibility with target was discussed in [24, 25] (latter of which introduces algorithms to learn better aligned kernels), by introducing an "alignment" metric defined by:

$$A(K_1(x,x'),K_2(x,x')) = \frac{\langle K_1(x,x'),K_2(x,x')\rangle_p}{\sqrt{\langle K_1(x,x'),K_1(x,x')\rangle_p \langle K_2(x,x'),K_2(x,x')\rangle_p}}, \tag{142}$$

where $\langle k_1(x,x'),k_2(x,x')\rangle_p = \int dp(x)dp(x')k_1(x,x')k_2(x,x')$ denotes the inner product of two bi-dimensional functions with respect to the data distribution $p(x)$. The so-called "kernel-target alignment" between a kernel $K(x,x')$ and target function $\bar{f}(x)$ is then defined by $A(K(x,x'),\bar{f}(x)\bar{f}(x'))$.

To get further insight about the differences between the kernel alignment metric and our task-model alignment, we can evaluate the kernel alignment metric using the eigendecompositions of the kernel and the target function. We obtain:

$$A(K(\mathbf{x},\mathbf{x}'),\bar{f}(\mathbf{x})\bar{f}(\mathbf{x}')) = \frac{1}{\sum_\rho \eta_\rho \bar{w}_\rho^2}\frac{\sum_\rho \eta_\rho^2 \bar{w}_\rho^2}{\sqrt{\sum_\rho \eta_\rho^2}}, \tag{143}$$

which is a scalar between $[0,1]$. In contrast, the task-model alignment states that when the cumulative power $C(\rho) = \frac{\sum_{\rho'\leq\rho}\eta_{\rho'}\bar{w}_{\rho'}^2}{\sum_{\rho'}\eta_{\rho'}\bar{w}_{\rho'}^2}$ at each mode ρ for a target functions is entry-wise larger than the $C(\rho)$ for another target function, the target function with larger $C(\rho)$ is learned with less training data then the other. While the cumulative power contains information about each learning stage ρ, kernel alignment is an aggregate measure of how the kernel and task is aligned.

To study the similarities between our prediction and [24] in a simple context, let us consider the regression task with the kernel $K(x,x') = \bar{f}(x)\bar{f}(x')$ and target $\bar{f}(x)$. This kernel and the target are perfectly aligned under the metric of [24] (A=1). The kernel has only a single eigenfunction $\phi(x) = \bar{f}(x)/\sqrt{\langle \bar{f}^2(x)\rangle_p}$ and hence a single non-zero eigenvalue $\eta = \langle \bar{f}^2(x)\rangle_p$. Furthermore, the eigenspace expansion of the target function gives trivially $\bar{w} = 1$. Hence, all the target power is placed in the first eigenfunction. Our theory implies that the $\bar{f}$ is the target function whose generalization error falls fastest under this kernel ($C(1) = 1$). Interestingly, in this case the generalization error has the same form as the band-limited case.

Relation to Other Statistical Physics Approaches to Kernel Machines

In the statistical physics domain, the replica method has been used to calculate classification learning curves for support vector machines when the data distribution is spherically symmetric and high dimensional, and for a specific class of target functions [26, 27]. These works revealed a countably infinite number of consistent thermodynamic limits: $P, D \to \infty$ with $P = \alpha D^L$ for integer L, which arise due to the degeneracy of kernel eigenvalues for all spherical harmonics of degree L. In the L-th learning stage, polynomials of degree L are being estimated as α increases. The rotation invariant kernels we discuss in this paper show the same learning stages, although for kernel regression rather than kernel SVM. However, our theory not only applies to spherically symmetric and infinite dimensional settings but is also shown to work for more general data distributions and a wide range of target functions, including realistic datasets such as MNIST and CIFAR. To aid the study of kernel methods in these more general distributions, we introduce useful metrics such as cumulative power $C(\rho)$ to quantify the alignment of the kernel with the learning task of interest.

The generalization of regularized linear regression with isotropic Gaussian features was analyzed with the replica method in [28] and diagrammatic methods in [29, 30, 31]. These works predicted learning curves equivalent to a special case of our kernel regression theory: the white band-limited spectrum. In this special case, we provide a phase diagram showing how effective noise and regularization can alter the non-monotonic behavior of the learning curve. We further show the universality of this learning curve and phase diagram to any setting where the kernel admits a truncated Mercer decomposition with all eigenvalues equal. We explicitly illustrate such an equivalence in the spherically symmetric data setting. In the L-th learning stage ($P \approx D^L$ with $D \to \infty$), any kernel essentially reduces to white band-limited kernel with $N(D, L)$ equal variance modes (spherical harmonics of degree L). We show that the higher degree components of the target act as effective noise while higher degree kernel eigenvalues act as effective regularization, allowing the phase diagram from the white band-limited setting to carry over to this interesting case.

Generalization error for Gaussian process regression, where the target function is a random field with covariance kernel K_t, was analyzed in the average case by Peter Sollich [32, 14]. Using the Sherman-Morrison inverse formula, he derived a continuous approximation of the learning curves from a partial differential equation for κ. In his work, he introduced a variety of approximations to learning curves based on this PDE approach. The learning curves obtained in the present work with the replica method agree with his *lower continuous approximation* in the kernel regression limit. Our theory goes beyond this work both in formalism and the results. Our framework allows the computation of other relevant observables (training error, average estimator, bias, and variance) by simply including additional sources in our partition function. We provide a detailed analysis of the generalization error expression to elucidate important heuristics for generalization and further show its applicability to real datasets.

Recent works on the effects of over-parameterization in random feature models have been investigated with the replica method [33, 16] and with random matrix theory tools [8, 34]. In the random features model, the last layer weights in a randomly initialized neural network are trained. In this setting the learning curves depend on the input dimension D, the number of random features N (network width) and the number of data points P. Typically, the authors consider fitting noisy linear target functions with these networks. In this model, two types of overfitting peaks can occur, one when $P \approx D$ and one when $P \approx N$ [35]. The relation between these peaks and the ones we see are discussed in the main text.

The equivalence between training infinite-width neural networks and kernel methods, allowed Cohen et al. to study the generalization of wide neural networks with a Gaussian field theory [6]. They obtained a different expression for the theoretical generalization error using a Poisson averaging technique, allowing them to expand their theoretical prediction in powers of $1/P$ at finite λ. We show in Supplementary Equation 64 that their expression for $\langle f^*(\mathbf{x}; P)\rangle_{\mathcal{D}}$ can be obtained from ours by a series expansion in a certain limit, showing that our result contains corrections not captured in their theory. Further, to the perturbative order they consider, they find that $\langle f^*(\mathbf{x}; P)^2\rangle_{\mathcal{D}} = \langle f^*(\mathbf{x}; P)\rangle_{\mathcal{D}}^2$, missing the variance of the estimator which plays a crucial role in our theory, especially for non-monotonicity. Furthermore, their expressions break down in the ridgeless limit and they resort to a renormalization approach to predict learning curves. Our expression is valid in this limit.

Our recent conference publication on kernel regression and the infinite-width limit of neural networks [12] laid the groundwork for the present investigation. However, the current paper significantly expands on it and brings in many new insights. First, the effect of label noise on generalization and the multiple descent phase diagram were not explored in our previous work which we provide an account of here. Second, we provide comparisons with CIFAR-10, not present in the previous work. Third, we introduced and analyzed the white band limited RKHS model. Fourth,

we emphasize task-model alignment as a heuristic here and provide a metric for it. Fifth, the field theory formalism presented in this paper is more general than the technique of [12], which only used the replica method to calculate the average of an inverse matrix. The flexibility of the new theory allowed us to compute other observables including training error, the expected estimator, and the covariance of the estimated coefficients over different datasets.

Supplementary References

[1] Carl Edward Rasmussen and Christopher K. I. Williams. *Gaussian Processes for Machine Learning (Adaptive Computation and Machine Learning)*. The MIT Press, 2005.

[2] Peter Lax. *Functional Analysis*. Wiley, 1966.

[3] Jorge Buescu. Positive integral operators in unbounded domains. *Journal of Mathematical Analysis and Applications*, 296(1):244–255, 2004.

[4] Arthur Jacot, Berfin Simsek, Francesco Spadaro, Clement Hongler, and Franck Gabriel. Kernel alignment risk estimator: Risk prediction from training data. In H. Larochelle, M. Ranzato, R. Hadsell, M. F. Balcan, and H. Lin, editors, *Advances in Neural Information Processing Systems*, volume 33, pages 15568–15578. Curran Associates, Inc., 2020.

[5] Ben Adlam and Jeffrey Pennington. The neural tangent kernel in high dimensions: Triple descent and a multi-scale theory of generalization. In *International Conference on Machine Learning*, pages 74–84. PMLR, 2020.

[6] Omry Cohen, Or Malka, and Zohar Ringel. Learning curves for deep neural networks: a gaussian field theory perspective. *arXiv preprint arXiv:1906.05301*, 2019.

[7] Mikhail Belkin, Daniel Hsu, Siyuan Ma, and Soumik Mandal. Reconciling modern machine-learning practice and the classical bias–variance trade-off. *Proceedings of the National Academy of Sciences*, 116(32):15849–15854, 2019.

[8] Song Mei and Andrea Montanari. The generalization error of random features regression: Precise asymptotics and double descent curve. *arXiv preprint arXiv:1908.05355*, 2019.

[9] Preetum Nakkiran, Gal Kaplun, Yamini Bansal, Tristan Yang, Boaz Barak, and Ilya Sutskever. Deep double descent: Where bigger models and more data hurt. In *8th International Conference on Learning Representations, ICLR 2020, Addis Ababa, Ethiopia, April 26-30, 2020*. OpenReview.net, 2020.

[10] Zhidong Bai and Jack W Silverstein. *Spectral analysis of large dimensional random matrices*, volume 20. Springer, 2010.

[11] Madhu S. Advani, Andrew M. Saxe, and Haim Sompolinsky. High-dimensional dynamics of generalization error in neural networks. *Neural Networks*, 132:428–446, 2020.

[12] Blake Bordelon, Abdulkadir Canatar, and Cengiz Pehlevan. Spectrum dependent learning curves in kernel regression and wide neural networks. In *Proceedings of the 37th International Conference on Machine Learning*. PMLR, 2020.

[13] Peter Sollich. Gaussian process regression with mismatched models. In T. Dietterich, S. Becker, and Z. Ghahramani, editors, *Advances in Neural Information Processing Systems*, volume 14, pages 519–526. MIT Press, 2002.

[14] Peter Sollich and Anason Halees. Learning curves for gaussian process regression: Approximations and bounds. *Neural computation*, 14(6):1393–1428, 2002.

[15] Arthur Jacot, Berfin Simsek, Francesco Spadaro, Clement Hongler, and Franck Gabriel. Implicit regularization of random feature models. In Hal Daumé III and Aarti Singh, editors, *Proceedings of the 37th International Conference on Machine Learning*, volume 119 of *Proceedings of Machine Learning Research*, pages 4631–4640. PMLR, 13–18 Jul 2020.

[16] Stéphane d'Ascoli, Maria Refinetti, Giulio Biroli, and Florent Krzakala. Double trouble in double descent : Bias and variance(s) in the lazy regime. In *Proceedings of the 37th International Conference on Machine Learning*. PMLR, 2020.

[17] Arthur Jacot, Franck Gabriel, and Clément Hongler. Neural tangent kernel: Convergence and generalization in neural networks. In *Advances in neural information processing systems*, pages 8571–8580. Curran Associates, Inc., 2018.

[18] Sanjeev Arora, Simon S Du, Wei Hu, Zhiyuan Li, Russ R Salakhutdinov, and Ruosong Wang. On exact computation with an infinitely wide neural net. In *Advances in Neural Information Processing Systems*, pages 8139–8148. Curran Associates, Inc., 2019.

[19] Jaehoon Lee, Lechao Xiao, Samuel Schoenholz, Yasaman Bahri, Roman Novak, Jascha Sohl-Dickstein, and Jeffrey Pennington. Wide neural networks of any depth evolve as linear models under gradient descent. In *Advances in neural information processing systems*, pages 8570–8581. Curran Associates, Inc., 2019.

[20] Mario Geiger, Arthur Jacot, Stefano Spigler, Franck Gabriel, Levent Sagun, Stéphane d' Ascoli, Giulio Biroli, Clément Hongler, and Matthieu Wyart. Scaling description of generalization with number of parameters in deep learning. *Journal of Statistical Mechanics: Theory and Experiment*, 2020(2):023401, Feb 2020.

[21] Feng Dai and Yuan Xu. *Approximation Theory and Harmonic Analysis on Spheres and Balls*. Springer New York, 2013.

[22] Anthony Ralston and Philip Rabinowitz. *A first course in numerical analysis.* Courier Corporation, 2001.

[23] Roman Novak, Lechao Xiao, Jiri Hron, Jaehoon Lee, Alexander A. Alemi, Jascha Sohl-Dickstein, and Samuel S. Schoenholz. Neural tangents: Fast and easy infinite neural networks in python. In *International Conference on Learning Representations*. OpenReview.net, 2020.

[24] Nello Cristianini, John Shawe-Taylor, André Elisseeff, and Jaz Kandol a. On kernel-target alignment. In T. Dietterich, S. Becker, and Z. Ghahramani, editors, *Advances in Neural Information Processing Systems*, volume 14, pages 367–373. MIT Press, 2002.

[25] Corinna Cortes, Mehryar Mohri, and Afshin Rostamizadeh. Algorithms for learning kernels based on centered alignment. *The Journal of Machine Learning Research*, 13(1):795–828, 2012.

[26] Rainer Dietrich, Manfred Opper, and Haim Sompolinsky. Statistical mechanics of support vector networks. *Phys. Rev. Lett.*, 82:2975–2978, Apr 1999.

[27] M. Opper and R. Urbanczik. Universal learning curves of support vector machines. *Phys. Rev. Lett.*, 86:4410–4413, May 2001.

[28] Madhu Advani and Surya Ganguli. Statistical mechanics of optimal convex inference in high dimensions. *Phys. Rev. X*, 6:031034, Aug 2016.

[29] J A Hertz, A Krogh, and G I Thorbergsson. Phase transitions in simple learning. *Journal of Physics A: Mathematical and General*, 22(12):2133–2150, Jun 1989.

[30] A Krogh and J. Hertz. Generalization in a linear perceptron in the presence of noise. *Journal of Physics A: Mathematical and General*, 25:1135, 01 1999.

[31] Peter Sollich. Finite-size effects in learning and generalization in linear perceptrons. *Journal of Physics A: Mathematical and General*, 27(23):7771, 1994.

[32] Peter Sollich. Learning curves for gaussian processes. In *Advances in neural information processing systems*, pages 344–350. MIT Press; 1998, 1999.

[33] Federica Gerace, Bruno Loureiro, Florent Krzakala, Marc Mezard, and Lenka Zdeborova. Generalisation error in learning with random features and the hidden manifold model. In Hal Daumé III and Aarti Singh, editors, *Proceedings of the 37th International Conference on Machine Learning*, volume 119 of *Proceedings of Machine Learning Research*, pages 3452–3462. PMLR, 13–18 Jul 2020.

[34] Trevor Hastie, Andrea Montanari, Saharon Rosset, and Ryan J. Tibshirani. Surprises in high-dimensional ridgeless least squares interpolation. *arXiv preprint arXiv:1903.08560*, 2019.

[35] Stéphane d'Ascoli, Levent Sagun, and Giulio Biroli. Triple descent and the two kinds of overfitting: Where and why do they appear? In *Advances in Neural Information Processing Systems*. Current Associates, Inc., 2020.
